# Arthropods and other biota associated with the Azorean trees and shrubs: *Laurusazorica* (Seub) Franco (Magnoliophyta, Magnoliopsida, Laurales, Lauraceae)

**DOI:** 10.3897/BDJ.10.e80088

**Published:** 2022-05-10

**Authors:** Noelline Tsafack, Rosalina Gabriel, Rui B. Elias, Mário Boieiro, Maria Teresa Ferreira, Paulo A. V. Borges

**Affiliations:** 1 cE3c - Centre for Ecology, Evolution and Environmental Changes / Azorean Biodiversity Group & CHANGE – Global Change and Sustainability Institute, University of the Azores, Faculty of Agricultural Sciences and Environment, Rua Capitão João D` Ávila, São Pedro, 9700-042, Angra do Heroísmo, Azores, Portugal cE3c - Centre for Ecology, Evolution and Environmental Changes / Azorean Biodiversity Group & CHANGE – Global Change and Sustainability Institute, University of the Azores, Faculty of Agricultural Sciences and Environment, Rua Capitão João D` Ávila, São Pedro, 9700-042 Angra do Heroísmo, Azores Portugal; 2 Regional Secretariat of Environment and Climate Change, Project LIFE BEETLES (LIFE 18 NAT/PT/000864), Rua do Galo n. 118, 9700-040, Angra do Heroísmo, Azores, Portugal Regional Secretariat of Environment and Climate Change, Project LIFE BEETLES (LIFE 18 NAT/PT/000864), Rua do Galo n. 118, 9700-040 Angra do Heroísmo, Azores Portugal

**Keywords:** *Laurusazorica*, Azores, islands, native forest, arthropods, vascular plants, bryophytes, liverworts, mosses, lichen, endemic, native and introduced species

## Abstract

This study explores the composition and structure of species communities associated with the native Azorean tree species *Laurusazorica* (Seub) Franco (Magnoliophyta, Magnoliopsida, Laurales, Lauraceae). Communities were sampled in six Islands covering the occidental (Flores), central (Faial, Pico, Terceira) and eastern (São Miguel, Santa Maria) groups of Azores Archipelago during the BALA project, using standardised sampling protocols for surveying canopy arthropod fauna. In addition, the study characterises the distribution of species regarding their colonisation status and feeding modes and, finally, compares communities of different Islands.

Ninety-four arthropod species totalling 10,313 specimens were collected on *L.azorica*. The Arthropod community was dominated by Hemiptera species, most of them being herbivores. Endemic and native species showed a very high abundance representing about 94% of the total species abundance. However, despite introduced species being represented by few individuals (6% of the total abundance), their diversity was remarkable (28 species and no significant difference with diversity found in endemic and native species communities). Analysis of rarity patterns revealed a stable community of endemic species (alpha gambin SAD model approaching a log-normal shape), intermediate stable community of native species (alpha SAD gambin model approaching a poisson log-normal) and a less stable community of introduced species (alpha SAD gambin model approaching a log-series shape). A dissimilarity analysis revealed high similarity between communities of Terceira and Pico and high dissimilarity between Flores and Faial communities. We observed a clear individualisation of the different islands when considering endemic species, whereas we observed high overlap when considering native and introduced species groups. Canopy community distribution confirms the results obtained in a previous study which suggest the stability of native and endemic arthropods species communities over introduced species community in native forests fragments.

Arthropod species were richer than bryophytes, lichens and vascular plants species. We found that *L.azorica* serve as the substrate for very few vascular plants species (four epiphytes species), which were present in all Islands, except *Elaphoglossumsemicylindricum*, which does not occur in Santa Maria. *L.azorica* shelters a significant number of bryophytes and lichens species. Thirty-two lichens and 92 bryophyte species, including 57 liverworts and 35 mosses, are referred to this phorophyte. Five bryophyte species, all Azorean endemics, are considered Endangered by IUCN Criteria. *L.azorica* harbours a poor community of epiphyte vascular plant species and all of them were ferns, but the community of bryophytes and lichens are not negligible although very low compared to the community found on other previously studied Azorean trees, the Azorean cedar *Juniperusbrevifolia*.

The present study shows that most islands present particular species distribution patterns without geographical correlation and that conservation programmes should be adapted to each Island. The study, therefore, calls for a specialisation of conservation programmes for each of the Islands.

## Introduction

Forest canopy represents the space between the soil and the atmosphere. Ulyshen (2011) defined the habitat as all the aboveground plant structures and the interstitial spaces between them. Forest canopies are, therefore, characterised by their vertical structure that offer substrate, resources and shelter for a large range of species (Schowalter and Ganio 1998).

The difficulties in reaching forest canopy for sampling have long restricted the number of studies investigating biodiversity in this habitat (Nadkarni 1994). Thanks to new sampling techniques (Mitchell et al. 2002, Schowalter and Chao 2021) and the interest of forest conservationists, the number of studies investigating species communities of forest canopy is increasing with emphasis on arthropods (Kelly and Southwood 1999, Valencia-Cuevas and Tovar-Sánchez 2015, Vaca-Sánchez et al. 2021), but also other biota (Nadkarni and Solano 2002, Patiño et al. 2018). Knowledge on how the forest canopy matrix supports species communities helps to optimise conservation programmes (Southwood and Kennedy 1983, Krüger and McGavin 2000, Ribeiro et al. 2005, Müller and Goßner 2007, Stahlheber 2016), with the recent recognition that the canopies can harbour a higher number of rare species of wood-inhabiting beetles in northern Europe (Haack et al. 2022).

Several studies showed the exceptional richness of arthropod communities in Azorean native forests (Borges et al. 2008, Cardoso et al. 2009, Rego et al. 2019, Florencio et al. 2021), as well as their vulnerability because of land use intensification, pressure with intensive management and climatic changes (Triantis et al. 2010, Terzopoulou et al. 2015, Ferreira et al. 2016). Additionally, recent studies also showed the crucial importance of native forest for endemic and native non-endemic species (Borges et al. 2008, Cardoso et al. 2009, Triantis et al. 2010, Meijer et al. 2011, Florencio et al. 2013, Tsafack et al. 2021b). Yet, few studies have explored Azorean arthropod communities at fine grain scale, especially the contribution of endemic tree species to species communities that have been under-investigated (but see Florencio et al. 2013, Rego et al. 2019).

It is in that context that we decided to fill the gap in Azores. We planned to investigate arthropod and plant communities associated with six main endemic tree species. We organised our investigation in a series of six studies. A first publication explored species communities associated with the Azorean cedar *Juniperusbrevifolia* (Hochst. ex Seub.) Antoine (Nunes et al. 2015). Four other studies will follow the current study and will present our investigation of species communities associated with, respectively, *Ilexazorica* Gand., *Ericaazorica* Hochst. ex Seub; *Vacciniumcylindraceum* Sm. and *Myrsineretusa* Aiton trees and shrub species.

The present study focuses on the Azorean endemic tree species *Laurusazorica* (Seub) Franco. The study characterises the distribution of species regarding their colonisation status and feeding modes and finally compares communities of six Azorean Islands covering the western (Flores), central (Faial, Pico, Terceira) and eastern (São Miguel, Santa Maria) groups. These studies are intended to be the baseline for future evaluations of the impacts of common biodiversity erosion drivers (e.g. habitat loss and degradation, invasive species, climatic changes) on the diversity or organisms associate with the canopy of Azorean endemic trees and shrubs.

## Materials and Methods

### The target tree species Laurusazorica (Seub.) Franco

**Figure 1.**
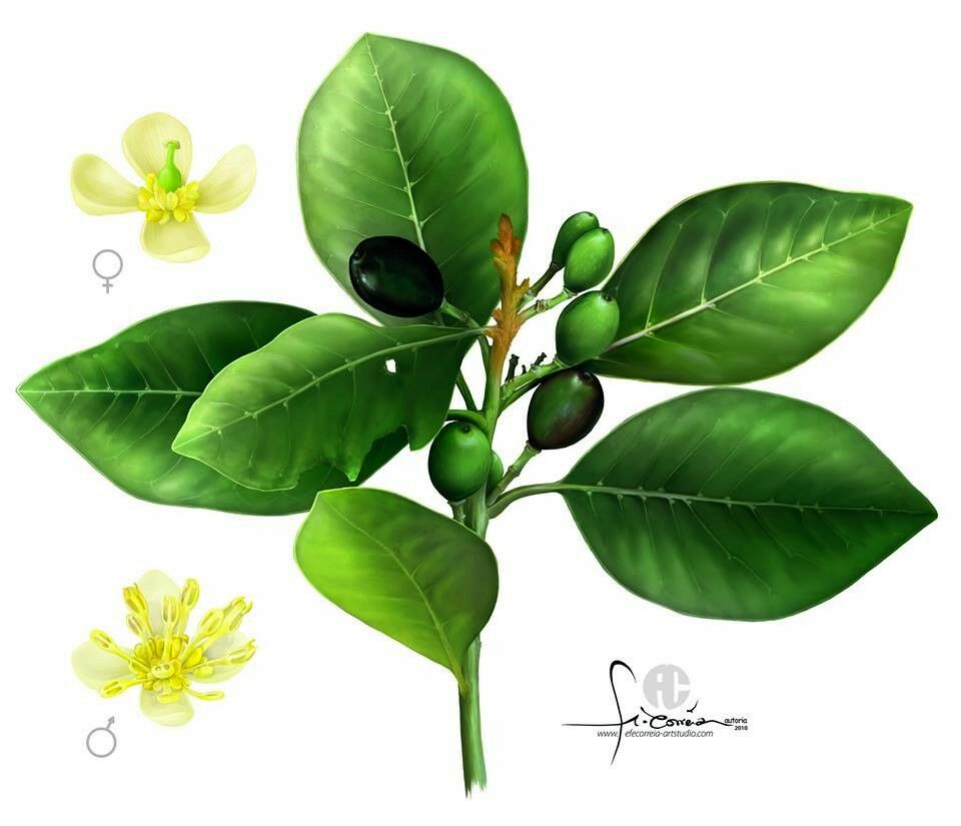
Twig of *Laurusazorica* (Seub.) Franco showing the leaves, unripe fruits (green) and ripe fruits (black). On the left are the details of the female and male flowers. Scientific illustration by Fernando Correia (www.efecorreia-artstudio.com). With permission of Azorina – S.A.

*L.azorica* (Magnoliophyta, Magnoliopsida, Laurales, Lauraceae), the Azorean laurel, is a dioecious evergreen tree that grows up to 15 m height. The leaves (up to 15 cm long and 8 cm wide) are alternate, simple, entire, elliptic, oblong or obovate, acute and aromatic. Young twigs and leaves are brown-tomentose, becoming glabrous. Flowers are yellowish-green; perianth 4-lobed, segments ca. 4 mm. The fruits are fleshy, ellipsoid, up to 2 cm, black (when ripe) (Fig. 1) (Franco 1971, Schaefer 2005).

Endemic to the Azores, this species is common in submontane laurel forests and *Juniperus-Ilex* montane forests. Scattered or locally common in *Picconia-Morella* lowland forests, *Juniperus* montane woodlands and *Pittosporum* exotic forests (Elias et al. in press). It can also be found, rare or scattered, in *Cryptomeria* plantation forests. It occurs mainly between 100 and 900 m altitude, in all Azorean Islands.

Before Portuguese occupation and the process of forest cut for wood consumption and agriculture development, *L.azorica* was probably one of the most common tree species in the Azores. In fact, submontane laurel forests could have occupied more than 40% of the islands' surface and this species is also very frequent in montane forests that were the dominant vegetation between 600 and 900 m altitude in Faial, Pico, São Jorge, Terceira and São Miguel (Elias et al. 2016). However, landscape transformation by the Portuguese settlers affected mostly the laurel forests and *L.azorica* now occurs mostly in the remnant *Juniperus-Ilex* montane forests.

### Study sites

Data related to arthropods were obtained within the scope of the BALA project (Biodiversity of Arthropods in the Laurisilva of the Azores) that started in 1999 (Borges et al. 2005, Ribeiro et al. 2005). *Laurus* trees were selected and sampled in six Azorean Islands covering the western (Flores), central (Faial, Pico, Terceira) and eastern (São Miguel, Santa Maria) groups. The location map and an extensive description of Azorean Archipelago and vegetation can be found in Nunes et al. (2015). Experimental design can be found in detail in Borges et al. (2005) and Ribeiro et al. (2005). In summary, 100 transects of 150 m were sampled in seven Azorean Islands and 20 native forest reserves. In each transect, the three most frequent tree species were selected for sampling. We adopted a stratified sampling design: minimum of four transects for each forest fragment, but a different number of transects amongst Islands to reflect the area of forest reserves in the Islands. Considering the availability of *Laurus* trees, one forest fragment was monitored in Faial, two in Flores, five in Pico, six in São Miguel, three in Santa Maria and thirty-five in Terceira. For comparison between Islands, we standardised data to the number of samples available.

### Arthropod sampling and identification

Arthropods were sampled using a beating tray. We used a modified beating tray, which consisted of an inverted cloth funnel pyramid of 1 m wide and 60 cm deep. A plastic bag was placed at the tip where arthropods, leaves and small branches were collected (Ribeiro et al. 2005). A beating tray is an efficient method to assess species diversity, abundance and distribution. Species were sorted and identified using a Leica M5 stereomicroscope, specific literature and a reference collection on the Azorean terrestrial arthropod biodiversity. When identification was not possible, we kept a morphospecies identifier to a given taxon. The specimens were deposited in the Entomological Collection Dalberto Teixeira Pombo at the University of the Azores. Each species was assigned to one of the three colonising statuses according to its distribution in the Azorean Archipelago (Borges et al. 2010): endemic (species restricted to the Azores), native non-endemic (species that arrived naturally to the Archipelago, but are also present elsewhere) and introduced (species accidentally or deliberately introduced by man). Species details can be consulted in Borges et al. (2016).

### Lichens, bryophytes and vascular plants sampling

Two kinds of data were assessed for this inventory: literature records (Suppl. material 1) and herbarium records (Suppl. materials 2, 3, 4, 5). Most of the herbarium records were collected using standard collection protocols on native vegetation areas (Gabriel and Bates 2005, Borges et al. 2018) and others were collected for herborisation purposes. Samples were obtained from relevés with 30 cm or 10 cm-side, placed at different heights on the trees, allowing the estimation of cover and richness of species. Taxonomy for lichens follows Aptroot et al. (2010), while for bryophytes, taxonomy follows Hodgetts et al. (2020). All data are included in the Azores Bioportal (http://azoresbioportal.uac.pt/) for the general public.

More details on lichen and plants species sampling can be found in our previous work (Elias et al. 2019, Gabriel et al. 2019, Gabriel 2000). Vascular plants data, used in this study, result from the data collected by RBE and are listed in Suppl. material 2. The bryophytes and lichen dataset, used in this study, are listed in Suppl. materials 3, 4, 5 and are deposited in the Cryptogamic Collection of the Herbarium of the University of the Azores (AZU) (Angra do Heroísmo).

### Data analysis

We described and compared the structure and the composition of species communities of *L.azorica* in different islands. The present analysis design follows the analysis plan of our previous study on taxa associated with *Juniperusbrevifolia* (see Nunes et al. (2015) for more details). Since the sampling effort was different between islands (different number of transects available with *Laurus*), we standardised the data for comparison, but we used raw data when comparing different groups inside the same island.

Therefore, for species composition, we compared Islands for their species richness, abundance, functional groups and feeding modes and, for species community structure, we investigated patterns of rarity with species abundance distribution models and with species community similarity analyses.

#### Arthropods

For all analysis, we used the complete dataset including juveniles and adult specimens identified at the species and morphospecies level (Suppl. material 6). The taxonomy follows the most updated nomemclature recently posted in the AZORESBIOPORTAL (see https://azoresbioportal.uac.pt/)

**Species composition**

Each species was assigned to a functional group (predator, herbivore, saprophyte and fungivore) and a feeding mode (external digestion and sucking, chewing and cutting, piercing and sucking, siphoning, not feeding) (see Rigal et al. (2018) for details).

We compared the different islands for their species abundances and richness. Islands were also compared for species abundance and richness within the different trophic functional groups using the Kruskal-Wallis test and a pairwise Dunn test.

**Species community structure**

We explored rarity patterns of communities using Preston octaves and alpha-gambin values. Preston (1948) defined classes of abundance called octaves which are used to visualise the distribution of species in the community. Using gambin models, abundance octaves were created using a log2 base (1, 3, 7, 15, 31, 63.....) (Gray et al. 2006, Matthews et al. 2014). Following the quartile rule suggested by Gaston (1997)), species of the first 25% of octaves are considered as rare species.

We used unimodal gambin models to fit the species abundance distribution at the Archipelago level and in each Island separately. We compared gambin models shapes and the value of the parameter α change between Island communities.

We used Non-metric Multidimensional Scaling (NMDS) to examine the similarity between the different island communities with the Bray-Curtis dissimilarity metric. Bootstrapping approaches were used to analyse the significance of NMDS ordination. First, a Permutational Multivariate Analysis of Variance was performed using the function *Adonis* to test if species communities were different between Islands. Second, an Analysis of Similarities (ANOSIM) was performed using the function *Anosim* to examine if the difference was significant. The higher Anosim R-value, the higher the dissimilarity between Islands.

In order to identify species that contributed the most to the dissimilarity observed between Islands, pairwise comparisons were performed using the function *Simper* with 999 permutations with the Bray-Curtis distance. This analysis also allows us to assess the significance of species contributions.

All analyses were run using the R programme (R Core Team 2021). To fit gambin models, we used the function *fit_abundances* in the “gambin” library (Matthews et al. 2014). The functions *Adonis, Anosim* and *Simper* are implemented in the library “Vegan” (Oksanen et al. 2020).

#### Lichens, bryophytes and vascular plants

Vegetation data were analysed using a descriptive approach because datasets were not large enough to allow comparison between Islands (see Suppl. materials 1, 2, 3, 4, 5). Further information, regarding Establishment means (Gabriel et al. 2010) and IUCN criteria (Hodgetts et al. 2019) was also mentioned.

## Results

### Arthropods

**Species composition**

We collected a total of 10,313 specimens, corresponding to 94 species and morphospecies, 50 families, 13 orders and three classes (Table 1 and Suppl. material 6).

**Species richness**

**Figure 2.**
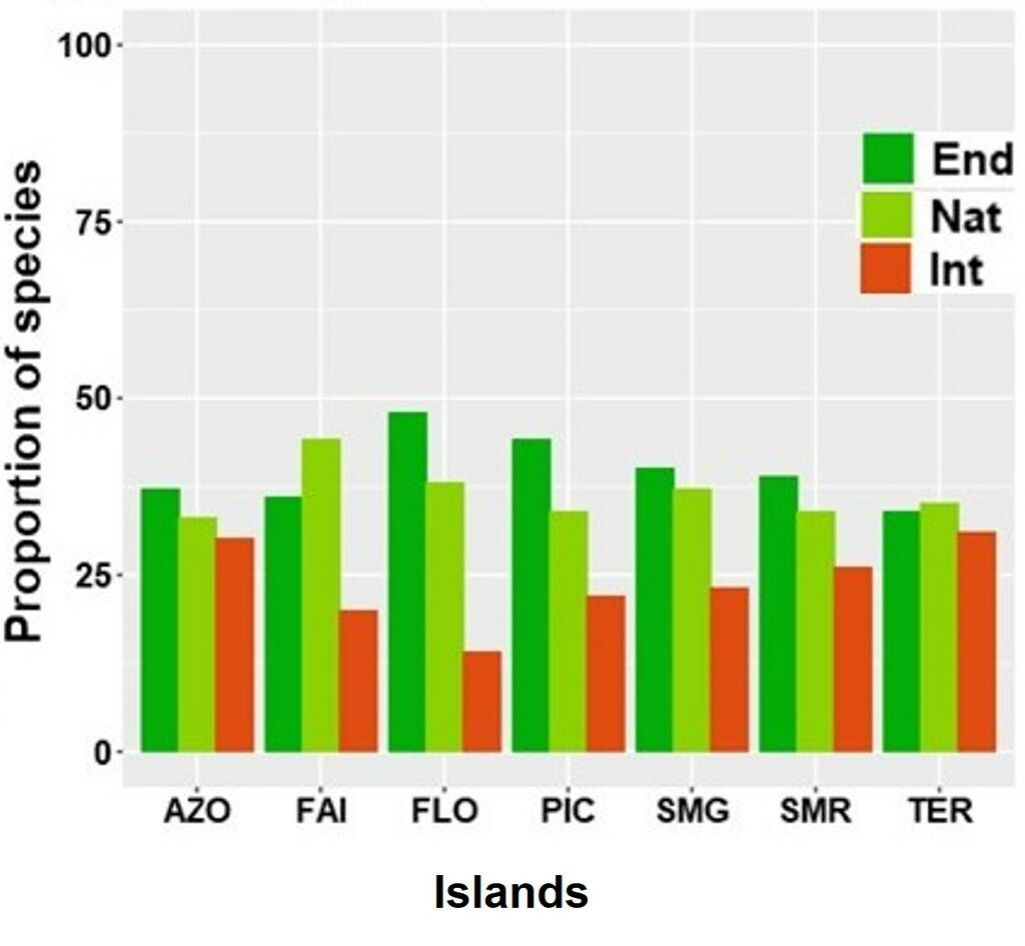
Proportion of overall arthropods species associated with *L.azorica* separately for the three colonising statuses: endemic (End), native (Nat) and introduced (Int) at Archipelago (AZO) and at the different Island level (FAI – Faial; FLO – Flores; PIC – Pico; SMG – São Miguel; SMR – Santa Maria; TER – Terceira).

Considering species richness of the different islands and in the Archipelago as a whole, we found that endemic, native and introduced species were evenly distributed and no difference was observed amongst the three groups regarding their number of species (Fig. 2, Table 2).

At Island level, we found that over 94 species observed at the Archipelago level and 71 species were collected in Terceira (Table 2). Standardising the sampling effort between Islands, we found that Santa Maria (SMR) was the most diverse Island, whereas São Miguel (SMG) was the least. The difference between SMR and SMG was significant (Suppl. material 7). The same pattern was observed within endemic and introduced species (Suppl. material 7). Regarding native species, a more complex pattern was observed: SMR was still the most diverse Island, but Flores (FLO) was the least and the difference was significant between the two Islands (Suppl. material 7).

**Figure 3.**
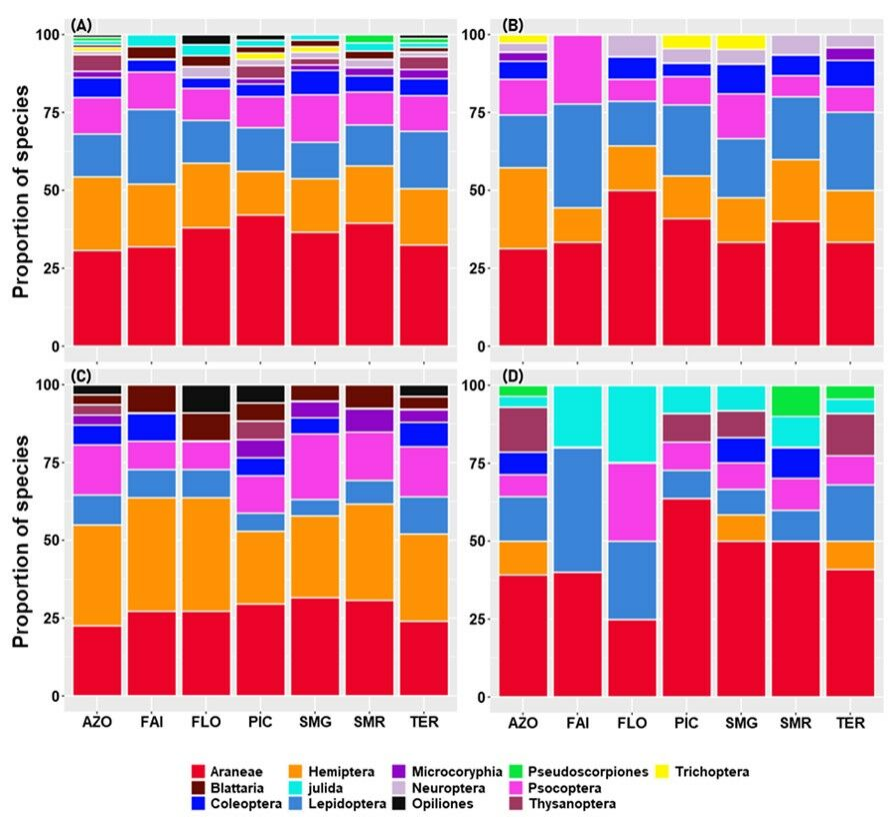
Proportion of arthropods species associated with *L.azorica* per order for all species (**A**) and for the three colonising status separately endemic (**B**), native (**C**) and introduced (**D**) species at Archipelago (AZO) and Island level (FAI – Faial; FLO – Flores; PIC – Pico; SMG – São Miguel; SMR – Santa Maria; TER – Terceira).

Species collected belong to thirteen orders. We found that most species were spiders (Araneae) at the Archipelago level and in almost every Island. This finding was true for the overall species as well as for the three colonising groups. Lepidoptera and Hemiptera were the second most rich groups (Fig. 3).

**Abundance**

**Figure 4.**
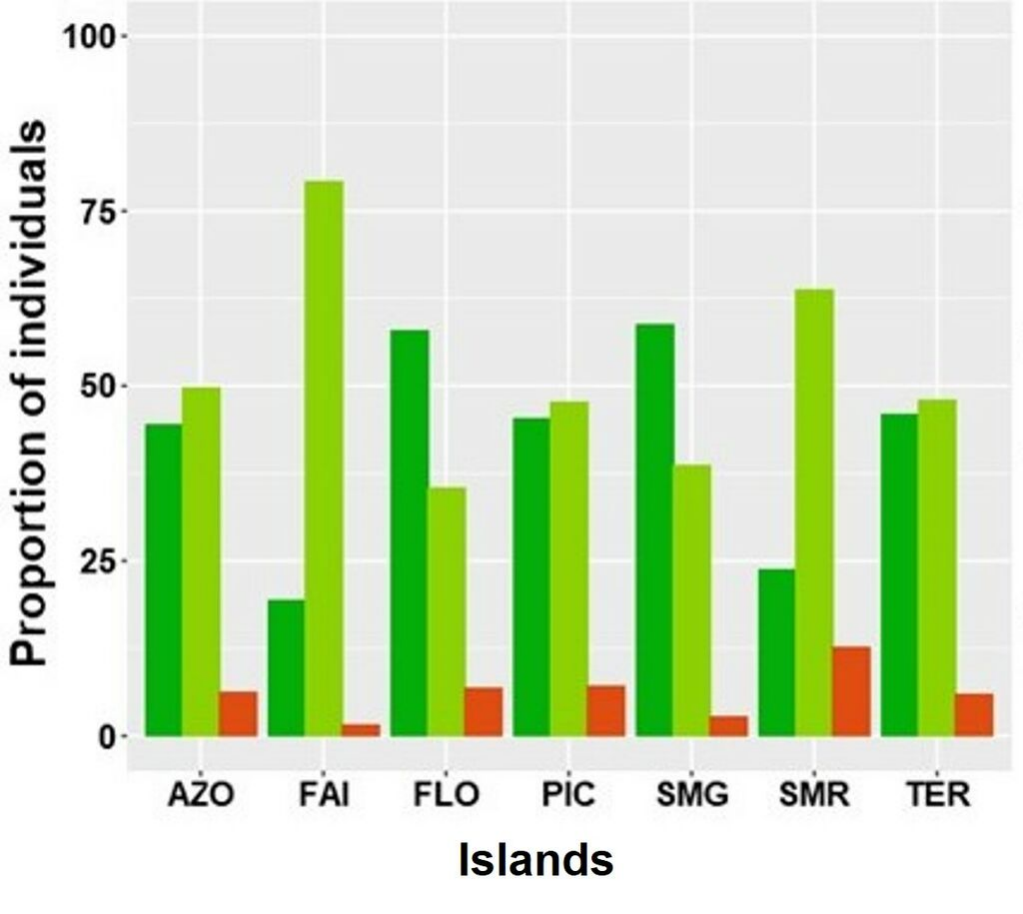
Specimens proportion of arthropods associated with *L.azorica* separately for the three colonising statuses: endemic (End), native (Nat) and introduced (Int) at Archipelago (AZO) and at the different Island level (FAI – Faial; FLO – Flores; PIC – Pico; SMG – São Miguel; SMR – Santa Maria; TER – Terceira).

Considering the Archipelago as a whole, most specimens were from native species (about 50%), endemic species accounted for 44% of collected individuals, whereas introduced species account for only 6% of the collected individuals (Fig. 4, Table 2). The same pattern is observed for Islands: introduced species comprised the least abundant group, while endemic and native represented about 90% of the total abundance and near 98% in Faial. In most Islands, native species was the most abundant group, except in FLO and in SMG where endemic species were most abundant (Fig. 4, Table 2). Species from the three colonising groups were significantly different (Table 2).

The six most abundant species have 50% of the total abundance: the native *Triozalaurisilvae* Hodkinson, 1990 (Hemiptera) (n = 2675); the endemic spider *Gibbaraneaoccidentalis* Wunderlich, 1989 (Araneae) (n = 662); the endemic moth *Argyresthiaatlanticella* Rebel, 1940 (Lepidoptera) (n = 510); the native spider *Lathysdentichelis* (Simon, 1883) (Araneae) (n = 498), the endemic spider *Savigniorrhipisacoreensis* Wunderlich, 1992 (Araneae) (n = 34) and the endemic tree hopper *Cixiusazoterceirae* Remane & Asche, 1979 (Hemiptera) (n = 426) (see details in Suppl. material 6). The top ten most abundant species include three more Hemiptera and a Blattaria all native or endemic.

Faial Island shows the highest number of specimens and Flores the lowest (Suppl. material 8). In addition, native species were more abundant in FAI than in the other Islands, but the difference was significant only between FAI and FLO (Suppl. material 9). The same pattern was observed when considering the total abundance (Suppl. material 9). Within endemic species, the significant difference was between SMG and SMR (Suppl. material 9) and within introduced species, the significant difference was between FAI and SMR (Suppl. material 9).

**Figure 5.**
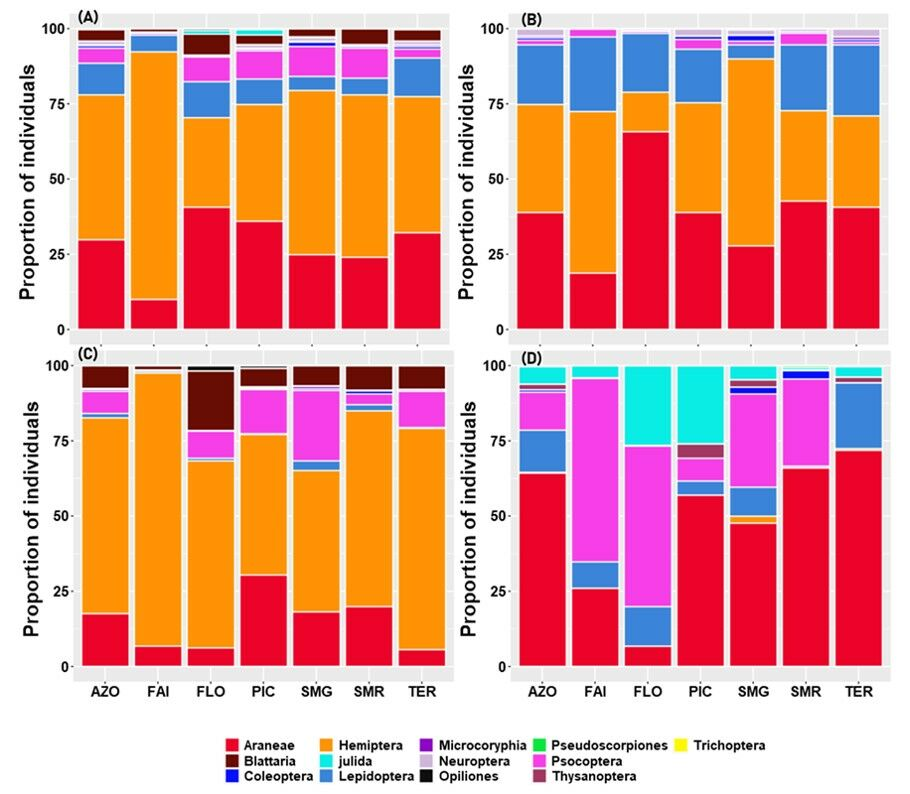
Abundance proportion of arthropods associated with *L.azorica* per order for all species (**A**) and for the three colonising statuses separately endemic (**B**), native (**C**) and introduced (**D**) species at Archipelago (AZO) and Island level (FAI – Faial; FLO – Flores; PIC – Pico; SMG – São Miguel; SMR – Santa Maria; TER – Terceira).

Considering the Archipelago as a whole (Fig. 5), Hemiptera were the most abundant group over all species (4958 specimens representing 48%) and also within native species (3316 specimens representing 65%), whereas Araneae were the most abundant group within introduced and endemic species, accounting respectively, for 64% and 39% (Fig. 5). Hemiptera were the second most abundant group (36%) within the endemic species, while they represented less than 1% of specimens within the introduced species. Amongst the introduced species, lepidopterans and booklice were the second and third most abundant species, representing 14% and 13%, respectively. Opiliones, Pseudoscorpiones, Julida, Coleoptera, Microcoryphia, Trichoptera and Thysanoptera altogether accounted for less than 1% for the total abundance and also within the different colonisation status groups, except Julida which represents 6% of the introduced species (Fig. 5).

At Island level, Hemiptera and Araneae account for more than 75% of species abundances in all species, endemic or native species groups. A different pattern was observed within introduced species; spiders, lepidopterans and booklice were the most abundant groups (Fig. 5). Although these patterns were globally observed in the different Islands, we found some differences. For example, in Flores, most of the introduced species were booklice and millipedes, while in Pico, they were spiders and millipedes (Fig. 5).

**Functional groups and feeding modes**

**Figure 6.**
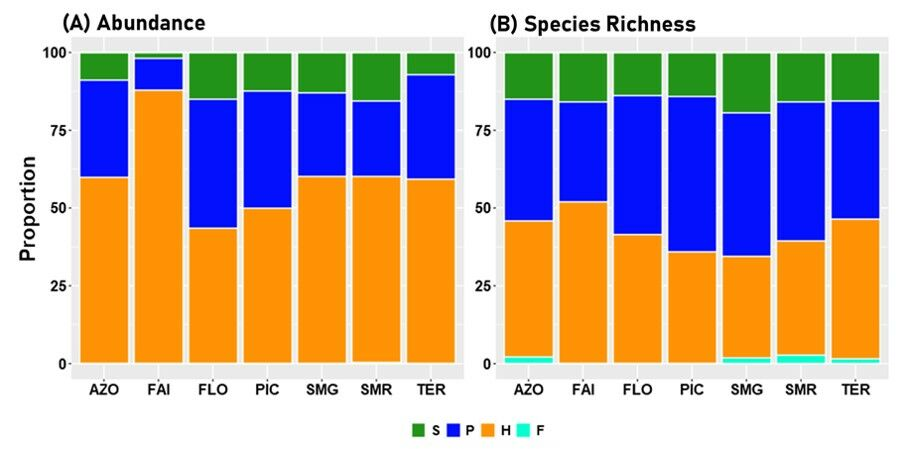
Abundance (**A**) and number of species (**B**) proportions of arthropods associated with *L.azorica* per different functional groups (S - saprophyte, P - predator; H - herbivore; F – fungivore) at Archipelago level (AZO) and Island level (FAI – Faial; FLO – Flores; PIC – Pico; SMG – São Miguel; SMR – Santa Maria; TER – Terceira).

Herbivores and predators represent about 80% of total species abundance and richness at the Archipelago level, as well as at the Island level. Herbivores were represented by 41 species (6159 specimens) and predators by 37 species (3225 specimens) (Fig. 6).

A comparison between Islands showed that abundance of herbivores per unit sample was higher in FAI and the difference was significant between FAI and FLO (Suppl. material 10). No differences were observed between abundances of predators in the different Islands, except between PIC and SMG. Saprophytes were more abundant in SMR and the difference was significant between SMR and FAI (Suppl. material 10). Fungivore species was the poorest group present only in three Islands SMR, SMG and Terceira (TER) and representing less than 1% species and abundance (two species and seven individuals, Suppl. material 8).

Crossing functional and colonising groups, we found that most herbivores were endemics and native species, while most predators were introduced species. The distribution pattern was observed both for abundance and species richness (Suppl. materials 11, 12).

**Figure 7.**
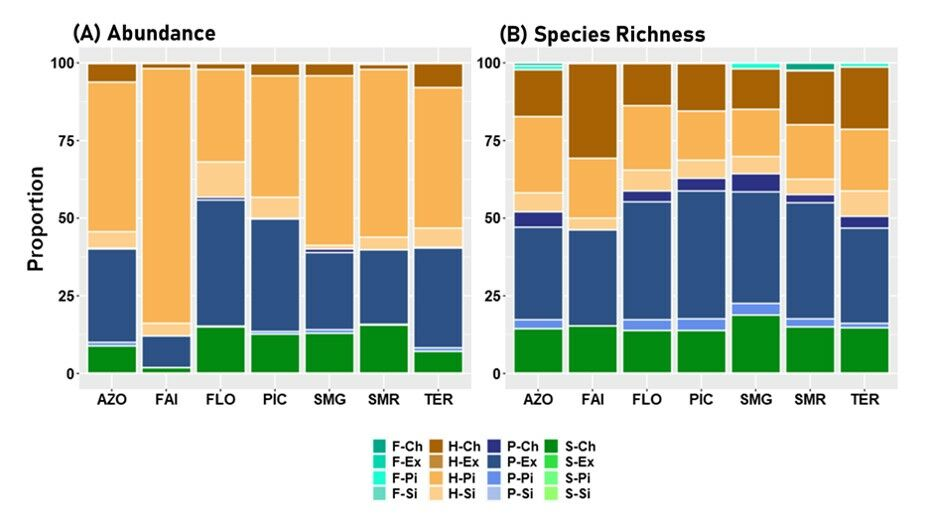
Abundance (**A**) and number of species (**B**) proportions of arthropods associated with *L.azorica* per different feeding modes at Archipelago (AZO) and Island level (FAI – Faial; FLO – Flores; PIC – Pico; SMG – São Miguel; SMR – Santa Maria; TER – Terceira). S - saprophyte, P - predator; H - herbivore; F – fungivore and Ex - external digestion and sucking; Ch - chewing and cutting; Pi - piercing and sucking; Si - Siphoning.

Species fall into four different feeding modes: external digestion and sucking; chewing and cutting; piercing and sucking; and siphoning. Very few species, 5% of the total species abundance (six species and 570 individuals), were siphoning species amongst them, five species were endemic species and one introduced species. Most individuals exhibited piercing and sucking feeding mode representing about 50% of the overall species abundance (5075 individuals). However, the chewing and cutting group was the most diverse group represented by 35 species (37% and 1581 individuals) (Fig. 7, Suppl. material 13).

Crossing analysis between feeding mode and colonising groups revealed a pattern similar to the functional group. Most endemic and native species were herbivores with piercing and sucking feeding mode and most introduced species were predators with external digestion and sucking (Suppl. materials 14, 15).

**Species community structure**

In this study, we considered rare species as those represented by seven individuals or less (see Gaston 1997). Thus, 45% of the overall species collected were considered rare. At the Archipelago level, about 79% of introduced species and 45% of native species were rare, while only 17% of endemic species were considered rare (Table 3). These proportions are highly variable between the different Islands, but the common pattern is that most of introduced species are rare, ranging from 70% in SMR to 100% in FAI (Table 3).

Overall, 13% of species were represented by only one individual (singleton). At the Archipelago level, about 29% of introduced species and 10% of native species were represented by only one individual, whereas only 3% of endemic species were singletons. The proportions of singletons species were variable between the different Islands and the higher proportions were found within introduced species ranging from 25% in FLO Island to 80% in FAI Island (Suppl. material 16). Suppl. material 16 also provides proportions of doubletons and tripletons species.

**Species abundance distribution patterns**

Preston’s abundances frequency distribution, also called octaves (Preston 1948), revealed interesting species distribution shapes.

**Figure 8.**
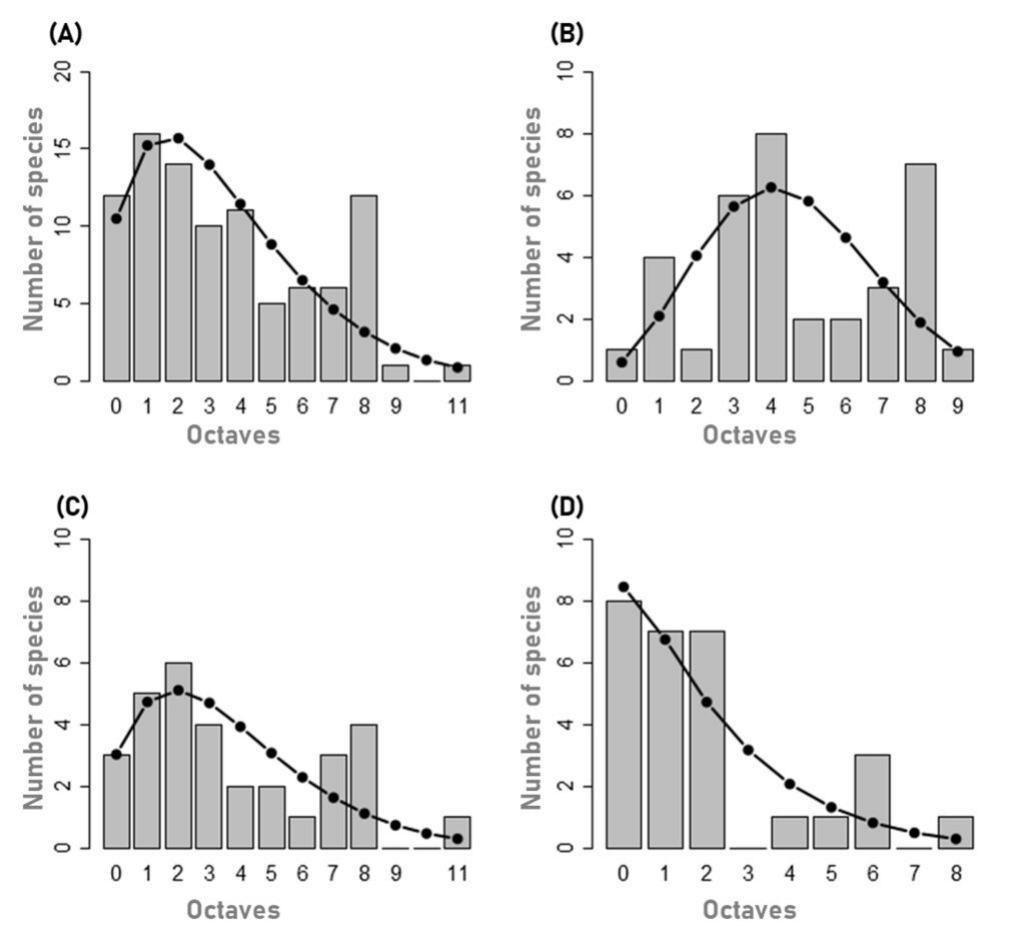
Species abundance distribution histograms for arthropods species communities associated with *L.azorica* collected in the Azores Archipelago with predicted values of the gambin models (black dots) for all species (**A**), endemic (**B**), native (**C**) and introduced (**D**) species. Graphs (B), (C) and (D) are scaled equally for the Y axis.

The overall assemblage at the Archipelago level, showed a poisson log-normal distribution shape (PLN) (A) with abundances distributed into 12 octaves and 45% of species falling in the first three octaves 0, 1, 2 and, therefore, considered rare species (Gaston 1997). This calculation method agrees with the manual method that we described in the section above. The native Hemiptera species *Triozalaurisilvae* Hodkinson falls in the octave 12 with 2675 individuals (Fig. 8).

Considering the three colonising groups, native species assemblage also showed the PLN shape Fig. 8C) and species abundances were distributed into 12 octaves. This method showed that about 45% of native species were rare species, falling into octaves 0, 1 and 2. The SAD model for endemic species (Fig. 8B) showed a log-normal shape (LN) with species distributed into 10 octaves and 17% of rare species. The Introduced group curve was a log-series (LS)-shape with species distributed into seven octaves and about 54% of rare species (Fig. 8D). Analysing species abundance distribution of the different Islands, we found variable patterns (Suppl. material 17). When considering the total species in Islands, gambin models shapes showed a PLN-shape in almost every Island, except in Faial which showed a LS-shape. An analysis of species separated in the different colonising groups revealed more details. SADs shapes of endemic species showed a PLN-shape in TER and SMR, but more LS-like in the other Islands. Conforming with native species at the Archipelago level, native species in Islands showed a LN-shape model, except in Faial where the model was more LS-shaped. SADs of introduced species showed a LS-shape in each Island, except in FLO where four species were collected and the four fall into different octaves, therefore presenting a quite flat gambin model.

**Figure 9.**
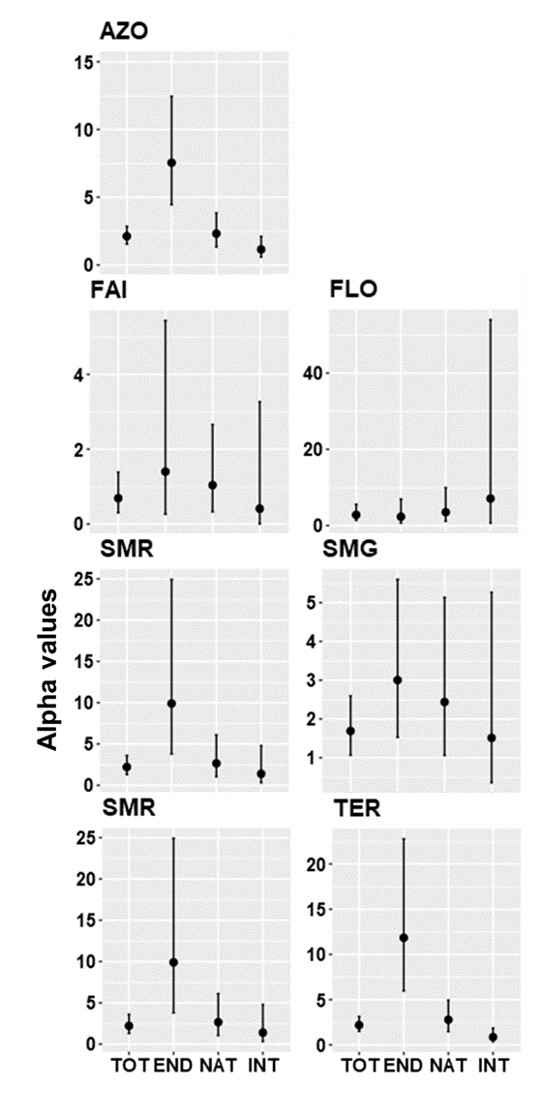
Alpha values of Gambin models for species distributions for arthropods species communities associated with *L.azorica* at Archipelago (AZO) and Island level (FAI – Faial; FLO – Flores; PIC – Pico; SMG – São Miguel; SMR – Santa Maria; TER – Terceira) for all species (TOT), endemic (END), native (NAT) and introduced (INT) species. Lines represent 95% confidence intervals.

Analysis of gambin α-values are consistent with the shapes of the different models. Considering the Archipelago level, endemic species group showed the highest α-value (7.55) followed by native (2.32) and introduced (1.15) species. The same pattern was observed in the different Islands, except in FLO, where the introduced species group has the highest α-value (7.08), followed by native (3.52) and endemic (2.33) species (Fig. 9, Table 4).

**Species community similarity**

**Figure 10.**
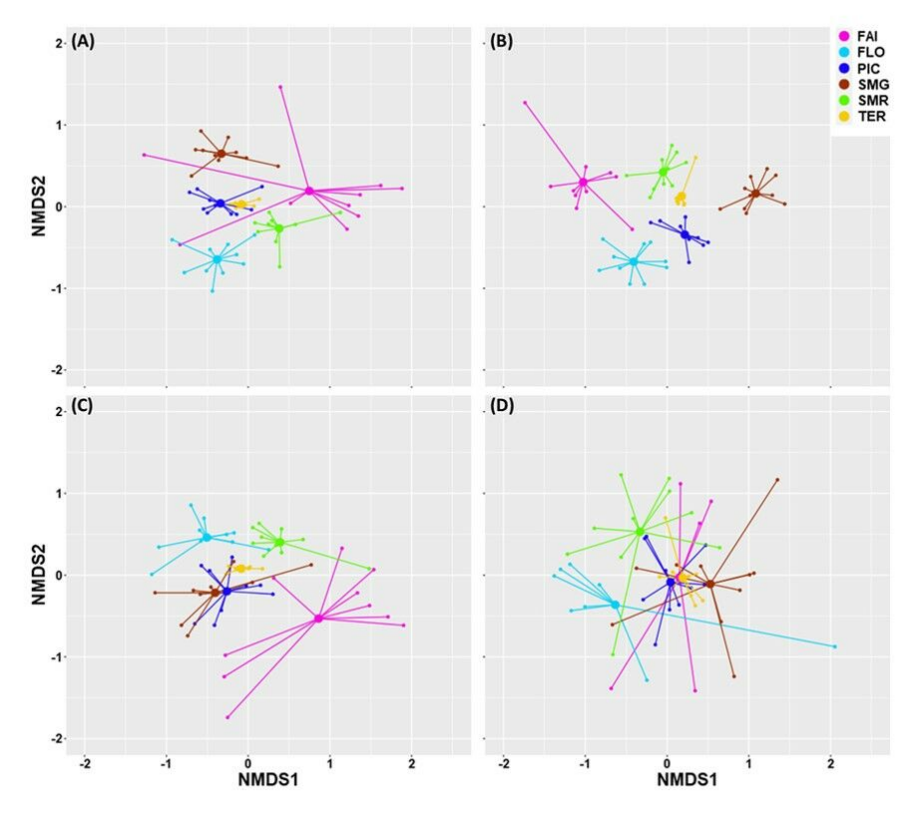
Non-metric Dimensional Scaling (NMDS) with Bray-Curtis dissimilarities for arthropod species communities, associated with *L.azorica* collected in the six Islands (FAI – Faial; FLO – Flores; PIC – Pico; SMG – São Miguel; SMR – Santa Maria; TER – Terceira) using: all species (A), endemic (B), native (C) and introduced (D) species with stress values, respectively 0.182, 0.194, 0.138 and 0.175.

We found high similarity between communities of Terceira (TER) and Pico (PIC) in one hand and, on the other hand, high dissimilarity between FLO and FAI (lowest (PIC-TER) and highest (FLO-FAI) Bray-Curtis index values) (Table 5). This result was not explicitly consistent with the number of shared species between the two Islands. Considering all species and native species, the highest number of shared species was between TER and SMG (46 and 17 species, respectively, for all species and native species). Within endemic species, the highest number of shared species was between TER and PIC (18 species) and between PIC and SMG (18 species). Within introduced species, the highest number of shared species was between TER and SMR (nine species). The Non-metric Multidimensional Scaling (NMDS) ordination shows Terceira species communities at the centre of the graphic with the other Islands displayed around Terceira (Fig. 10).

NMDS ordination also revealed that the different Islands were closer or more distant depending on whether we considered all species or species separated in different colonising statuses (Fig. 10A, B, C and D). Stress values of the NMDS ordination for the four investigated groups (all species, endemic, native and introduced) are very similar (respectively 0.182, 0.194, 0.138 and 0.175) (Fig. 10).

Considering all species: NMDS ordination (Fig. 10A) showed that species communities of PIC and TER were closer which is consistent with the lower dissimilarity index (value 0.50 in Table 5) observed between the two Islands. The graphical representation showed a high dissimilarity between FLO, SMG and SMR which is also confirmed by the high values of the Bray-Curtis index. Although two sampling points of FAI were close to FLO and SMG, the other points were scattered apart and the Bray-Curtis index values were very high ranging from 0.78 to 0.866 (Table 5).

Within endemic species, NMDS ordination (Fig. 10B) showed a clear distinct pattern indicating independent communities with low shared species amongst the different Islands. TER and SMR were the closest (index value = 0.560) and FAI and SMG the most distant (index value =0.91) (Table 5). NMDS ordinations of native species showed that FAI was apart from the other Islands (Fig. 10C). Bray-Curtis index values confirmed the ordination showing high values of indices ranging from 0.756 to 0.858 (Table 5). Contrasting the results of endemic and native species, ordination of introduced species shows high overlap between Islands (Fig. 10D). Islands sampling points were scattered and ravelled indicating the homogeneity of introduced species in the Archipelago (Fig. 10D).

ANOSIM sustained the structure observed in the different groups. Dissimilarities between the different Islands were all significant (P < 0.001) and R^2^ values indicated the highest dissimilarity within endemic species (R^2^ = 0.80), intermediate dissimilarity when considering all species (R^2^ = 0.66) or native species (R^2^ = 0.42) and very low dissimilarity within introduced species (R^2^ = 0.36).

### Bryophytes, lichens and vascular plants

**Vascular plants**

There are only four vascular plants, epiphytes of *L.azorica* and they are all ferns: *Hymenophyllumtunbrigense* (L.) Sm.; *Vandenboschiaspeciosa* (Willd.) G.Kunkel; *Elaphoglossumsemicylindricum* (T.E.Bowdich) Benl; Polypodiummacaronesicumsubsp.azoricum (Vasc.) Rumsey, Carine & Robba (see Suppl. material 2 for colonisation status and more details on their classification).

**Bryophytes**

We found that *L.azorica* was the substrate for both liverworts and mosses. Liverworts were represented by 57 species (three orders Jungermanniales, Metzgeriales and Porellales, comprising 18 families) (Suppl. material 3). Three species belong to the order Metzgeriales, while 28 species belong to Order Porellales (49%) and 26 to Order Jungermanniales (46%). Liverwort species may be found growing on living bark (epiphytic) or leaves (epiphyllic) and on decaying wood (epixylic). An important group of epiphyllous liverworts may be found growing on *L.azorica* leaves, including for instance: *Frullaniamicrophylla*, *Drepanolejeuneahamatifolia*, *Cololejeuneamicroscopica*, *Lejeunealamacerina*, *Myriocoleopsisminutissima*, *Metzgeriafurcata* and *Coluracalyptrifolia*. The Vulnerable *Cololejeuneaazorica*, may also colonise *L.azorica* leaves. A relatively small number of liverwort species has been found growing on decaying *L.azorica*, including *Plagiochilabifaria*, *Frullaniatamarisci* and *Telaraneaeuropaea*.

The 35 moss species belong to five orders (Bryales, Dicranales, Hookeriales, Hypnales and Orthotrichales). Most of species belong to order Hypnales (19 species; 54%), whereas Dicranales accounts for about a quarter of the species (nine species; 26%) (Suppl. material 4). Most of the species are epiphytes, including the Endangered *Daltonialindigiana*, *Echinodiumrenauldii* and *Thamnobryumrudolphianum*. It is interesting to note that *Hypnumuncinulatum* (Least Concern) and *Andoaberthelotiana* (Vulnerable) have been found growing on very old *L.azorica* leaves.

**Lichens**

The lichen community was composed of 32 species sorted into four classes (Arthoniomycetes, Dothideomycetes, Eurotiomycetes and Lecanoromycetes) and 13 identified orders (Arthoniales, Caliciales, Lecanorales, Monoblastiales, Ostropales, Peltigerales, Pertusariales, Pleosporales, Pyrenulales, Strigulales, Teloschistales, Trypetheliales, Verrucariales) (Suppl. material 5). Amongst lichens, four species are epiphyllous and the others are epiphytic.

## Discussion

A crucial point in a conservation programme is to accrue fine knowledge of species communities living in a particular system. In most ecosystems, the task is difficult, but thanks to their limited size and isolation, the probability to meet this fine grain knowledge in islands is high.

In the present study, carried out in Azores Islands, we explored arthropods and plant canopy species communities. We focused on species communities living on the endemic tree species *L.azorica*. We investigated the structure and composition of invertebrates and plants species community in six Azorean Islands covering the western (Flores), central (Faial, Pico, Terceira) and eastern (São Miguel, Santa Maria) Islands groups.

### Arthropods

**Communities species composition**

**Colonising status**

Arthropods species communities on *L.azorica* were dominated by native and endemic species. The ten most abundant species are all endemic or native (Suppl. material 6). The two groups were present in high proportion at the Archipelago level, but also at Island level, ranging from 87% (in S. Maria - SMR) to 98% (in Faial - FAI) of the total abundances. However, in terms of species richness, the number of introduced species was lower, but the difference between the three colonising groups was not significant. These findings support the results of a previous study on arthropods species associated with *Juniperusbrevifolia* canopies. Introduced species were represented by very few specimens (4%) and species (30%). The observed pattern might be explained by some characteristics of the *L.azorica* environment: (i) native forest: native species communities found in *L.azorica* benefit from the stability offered by native forest, whereas introduced species are hampered because they depend on disturbance-related factors (Borges et al. 2006, Borges et al. 2008, Florencio et al. 2013); (ii) habitat accessibility - the structural complexity of canopy might constrain the establishment of introduced species; (iii) niche saturation and species competition - the high diversity and abundance of canopy communities, as well as the dominance of predators species (e.g. Araneae, we developed this part below) on *L.azorica* contributed to saturation of ecological niches and, therefore, no places were left for introduced species and (iv) climate austerity - forest canopies, especially Azorean native forest canopies located at high elevations, are prone to climatic hazards including wind and rapid climate conditions turnovers. Some of these hypotheses have been developed in a previous study on *J.brevifolia* canopy (Nunes et al. 2015).

These results support those of a recent study in Azorean native forest, but whose samples were obtained with SLAM traps. Considering four dominant orders (Araneae, Coleoptera, Lepidoptera and Psocoptera), very few specimens of introduced species (6%) were collected, whereas the number of species was not different for endemic or native species groups (Tsafack et al. 2021b). A previous study, targeting both canopy and soil species of Azorean native forests, also found few specimens of introduced species (11%) compared to endemic and native species abundance (Gaspar et al. 2008). However, instead of no difference in species richness between the three biogeographical groups as we found, Gaspar et al. (2008) suggested the presence of high number of introduced species (34%) which is similar to our findings. The fact that the Gaspar et al. (2008) study included many plants and also soil samples might explain this discrepancy.

**Taxonomic composition**

Amongst the thirteen orders collected in this study, Hemiptera was the most abundant group and Araneae was the most diverse group. Together, Hemiptera and Araneae assemble more than 75% of abundances and more than 50% of number of species collected. However, within introduced species, Araneae was both the most abundant and the most diverse group. The ten most abundant species are mostly composed by Araneae and Hemiptera along with a moth and a cockroach (Suppl. material 6). The most abundant species *Triozalaurisilvae* Hodkinson, 1990 (Hemiptera) is considered a specialist of *L.azorica* (see Rego et al. 2019).

Our results support previous findings (Gaspar et al. 2008) which suggested that Araneae and Hemiptera were the most important groups in terms of number of species and abundance. However, contrary to our results, Hemiptera was the most diverse group and Araneae the most abundant group. In addition, Gaspar et al. (2008) results suggest that Coleoptera was the most diverse group in the canopy, whereas in the present study, very few beetles species (> 5%) were found on *L.azorica*. The differences might be explained by the fact that the current study is focused on the *L.azorica* tree, whereas Gaspar et al. (2008) included all trees in the native forests. The prevalence of Araneae and Hemiptera species was also observed on *J.brevifolia* canopies (Nunes et al. 2015).

**Functional and feeding groups**

Our results revealed the dominance of herbivores and predators species representing up to 80% of both number of species and number of specimens. The proportion of functional groups is consistent with the taxonomic composition that we previously developed. In fact, most of Hemiptera species being herbivores and all spider species being predators, the proportion of functional groups observed was then foreseeable. This is similar to functional groups observed on *J.brevifolia* (Nunes et al. 2015), on *Ericaazorica* (Ribeiro et al. 2005) and on other endemic tree canopies of Azorean Islands (Gaspar et al. 2008, Rego et al. 2019). Saprophytes accounted for about 5% of both abundance and number of species. Fungivores were represented by very few individuals (seven individuals) belonging to two species observed in three Islands (SMG, SMR and TER).

We found that this general pattern (dominance of herbivores tailed by predators and few saprophytes) was a common pattern observed in the different Islands. Moreover, the distribution of functional groups within the colonising groups was similar with some exceptions for the introduced taxa. The two fungivores species belong to the introduced taxa. We observed a dominance of saprophytes specimens in Flores (FLO) (> 50% of abundance).

Regarding species feeding mode, species communities found on *L.azorica* were mostly piercing and sucking species corresponding to species of the order Hemiptera and, therefore, representing about 50% of the overall species abundance (5075 individuals). Distribution patterns of species according to their feeding mode was closely related to their functional groups. Most endemic and native species were herbivores with piercing and sucking feeding mode, whereas most introduced species were predators with external digestion and sucking (Rigal et al. 2018).

**Community species structure**

Investigations on rarity and similarity patterns show that community structure at the Archipelago level contrast most of Islands communities’ structures. Some Islands, like Terceira (TER) and Santa Maria (SMR), showed high similarity with the study at the Archipelago level. However, for the other Islands, similarities with Archipelago level seem to depend on the colonising status of the species.

**Rarity patterns**

At the Archipelago level as well as for all Islands, a gambin model log-series (LS) shape fitted introduced species distribution, indicating that most of the introduced species were rare. LS-shape models are characteristic of simple species communities with dominance of rare species and very few represented by high number of individuals (Ulrich et al. 2016, Tsafack et al. 2021a). This finding is consistent with our investigations on species composition, where we found that, at Archipelago level, 79% of introduced species were rare. In some Islands like Faial, all introduced species (100%) were considered rare.

On the other side, gambin models fitting endemic and native species distributions fitted log-normal (LN) and poisson log-normal (PLN) shapes. LN distributions are generally considered describing stable communities (May 1975).

The present study suggests that introduced species communities are not yet well established on the *L.azorica* canopy which seems to be a stable refuge for indigenous (endemic and native) species communities.

**Similarity patterns**

Patterns of similarity between different Islands mainly rely on the colonising status, whether species were endemic, native or introduced species. Considering all species, no clear pattern emerged, communities of the six Islands overlapped, showing high similarities in species assemblages.

Contrary to communities collected on *Juniperusbrevifolia* (Ribeiro et al. 2005, Nunes et al. 2015), these were not observed as a clustering, mirroring the geographical distance between islands. At best, two Islands of the central group (PIC and TER) were closer, while the two Islands of the eastern group (SMG and SMR) were clearly distant. This stochastic overlap pattern was emphasised when we considered native and introduced species. This study highlights the high similarity on native species assemblages between Islands and, moreover, between introduced species. High similarity was also observed within introduced species collected on *J.brevifolia* (Nunes et al. 2015). However, as expected, endemic species assemblages offer a sharper distinct clustering between Islands. However, this pattern also does not rely on the geographical distance between Islands.

### Bryophytes, lichens and vascular plants

All species of vascular plants collected on *L.azorica* (four species) were epiphytes species and none was hemiparasites, contrasting the hemiparasite found on *J.brevifolia* (*Arceuthobiumazoricum* Wiens & Hawksw) (Nunes et al. 2015). One species was common to *L.azorica* and *J.brevifolia* (*Hymenophyllumtunbrigense* (L.) Sm).

*L.azorica* shelters a diverse community of lichen and bryophytes, but we found that species diversity was lower than on *Juniperusbrevifolia* community. This might be explained by the crinkled structure of *J.brevifolia* which offers diverse micro-habitats in all parts of the tree (Nunes et al. 2015). Contrary to *J.brevifolia, L.azorica* trunk and branches are rather smooth without cracks that favour retention of water and other nutrients which might serve as resources for arthropods or plants species. Furthermore, the pH values are quite different between the two phorophyte species and this is an important factor for discriminating different bryophyte communites (Gabriel and Bates 2005).

It is worth stressing that *L.azorica* supports a rich epiphyllous community, a feature which is characteristic of the Macaronesian mature forests, but very rare in other temperate habitats. More than 20 species have been found growing on *L.azorica*' leaves, adding a whole new layer of life to the Azorean forests.

About a fifth of the bryophytes, found associated with *L.azorica*, are IUCN conservation concern' species: seven mosses (three species, endangered; four species, vulnerable) and 12 liverworts (seven species endangered; five species vulnerable). Amongst the identified threats to the conservation of native ecosystems and species, both the habitat (Hodgetts et al. 2019) and climate change (Patiño et al. 2016) are important issues to avoid biodiversity erosion. Thus, the presence of epiphyllous bryophytes, which are relatively easy to recognise in the field, should be monitored as an indicator of habitat changes in the Azorean forests.

### Conclusions and implications for conservation

Our study identifies the contribution of the endemic tree *L.azorica* in supporting arthropods and plant species communities in native forest fragments. Although *L.azorica* seems to support poor communities compared to *J.brevifolia*, we found that profiles of species distribution provide clear insights on overall species distribution in native forest. Canopy community distribution confirms the results obtained in a previous study which suggest the stability of native and endemic species communities over introduced species community in native forests fragments (Tsafack et al. 2021b).

At the Azorean scale, the study warns again generalisations, suggesting that most Islands present a particular species distribution pattern without geographical correlation and that conservation programmes should be adapted to each Island.

## Supplementary Material

40381C07-2AB2-53F6-B9DA-4D1EC560CAEC 10.3897/BDJ.10.e80088.suppl1 Supplementary material 1 List of references Data type Table Brief description List of references used in the survey of mosses, liverworts (bryophytes) and lichens associated with *Laurusazorica*. File: oo_629541.docx https://binary.pensoft.net/file/629541 Rosalina Gabriel

9560B304-B189-50D6-8D3D-7F49CA7C8A2C 10.3897/BDJ.10.e80088.suppl2 Supplementary material 2 Vascular plants - division Pteridophyta
Data type Table Brief description List of vascular plants (Division Pteridophyta) associated with *Laurusazorica*. All species are present in the studied Islands with the exception of *Elaphoglossumsemicylindricum*, which does not occur on Santa Maria. Colonisation status for each species (Status) distinguishes amongst natives (NAT), Azorean endemics (END) and Macaronesian endemics (MAC). All Pteridophyta species are epiphytes. File: oo_629543.docx https://binary.pensoft.net/file/629543 Rui. B. Elias

B90D4ED5-8EBE-5F0D-A17E-2D0731BC2161 10.3897/BDJ.10.e80088.suppl3 Supplementary material 3 Bryophytes- division Marchantiophyta Data type Table Brief description List of liverworts (Marchantiophyta) associated with *Laurusazorica* in the various Azorean Islands. Records coming from literature (L), please check Supplementary Material 1 and/or from the Cryptogamic Collection of the Herbarium of the University of the Azores (AZU) (H). File: oo_629544.xlsx https://binary.pensoft.net/file/629544 Rosalina Gabriel

2232C3B5-E03E-54F0-9573-8A58952D5222 10.3897/BDJ.10.e80088.suppl4 Supplementary material 4 Bryophytes - division Bryophyta Data type Table Brief description List of mosses (Bryophyta) associated with *Laurusazorica* in the various Azorean Islands. Records coming from literature (L), please check Supplementary Material 1 and/or from the Cryptogamic Collection of the Herbarium of the University of the Azores (AZU) (H). File: oo_629545.xlsx https://binary.pensoft.net/file/629545 Rosalina Gabriel

37C9C341-EA90-5E40-8061-12545A598629 10.3897/BDJ.10.e80088.suppl5 Supplementary material 5 Lichens - division Ascomycota Data type Table Brief description List of lichens (Ascomycota) associated with *Laurusazorica* in the various Azorean Islands. Records coming from literature (L), please check Supplementary Material 17 and/or from the Cryptogamic Collection of the Herbarium of the University of the Azores (AZU) (H). All lichens are considered native in the Azores. File: oo_629546.xlsx https://binary.pensoft.net/file/629546 Rosalina Gabriel

C0581762-9F93-5542-8E78-5D0D22803A27 10.3897/BDJ.10.e80088.suppl6 Supplementary material 6 List of arthropod species associated with *Laurusazorica* in the Azores with abundance data per species and Island
Data type Table Brief description The classification system follows the general guidelines presented in Borges et al. (2010). The colonising status of each species is as follows: E – endemic; N – native; I – introduced. Islands coded as follows: FAI – Faial; FLO – Flores; PIC – Pico; SMG – São Miguel; SMR – Santa Maria; TER – Terceira. File: oo_626394.xlsx https://binary.pensoft.net/file/626394 Paulo A. V. Borges

8A26E260-F99F-52BF-947F-9FD32C7A7419 10.3897/BDJ.10.e80088.suppl7 Supplementary material 7 Species richness in Islands Data type Figure Brief description Comparison of standardised species richness values between the different Islands (FAI – Faial; FLO – Flores; PIC – Pico; SMG – São Miguel; SMR – Santa Maria; TER – Terceira) for all species (A), endemic (B), native (C) and introduced (D) species. Different letters indicate significant differences between Islands, based on Dunn’s multiple comparison test (p < 0.05). File: oo_629370.docx https://binary.pensoft.net/file/629370 Noelline Tsafack, Rosalina Gabriel, Rui. B. Elias, Mário Boieiro, Maria Teresa Ferreira, Paulo A. V. Borges

129D1AA4-E6C8-501D-9397-3FC6CB5CF4AC 10.3897/BDJ.10.e80088.suppl8 Supplementary material 8 Standardised values of abundance in the different Islands Data type Table Brief description Standardised values of abundance in the different Islands (FAI – Faial; FLO – Flores; PIC – Pico; SMG – São Miguel; SMR – Santa Maria; TER – Terceira) for all species (Total), endemic (END), native (NAT) and introduced (INT) species. File: oo_629374.docx https://binary.pensoft.net/file/629374 Noelline Tsafack, Rosalina Gabriel, Rui. B. Elias, Mário Boieiro, Maria Teresa Ferreira, Paulo A. V. Borges

2C4E9716-CC14-50CB-A6DB-CCFD6E6F917B 10.3897/BDJ.10.e80088.suppl9 Supplementary material 9 Abundance in Islands Data type Figure Brief description Comparison of standardised abundance values between the different Islands (FAI – Faial; FLO – Flores; PIC – Pico; SMG – São Miguel; SMR – Santa Maria; TER – Terceira) for all species (A), endemic (B), native (C) and introduced (D) species. Different letters indicate significant differences between Islands, based on Dunn’s multiple comparison test (p < 0.05). File: oo_629375.docx https://binary.pensoft.net/file/629375 Noelline Tsafack, Rosalina Gabriel, Rui. B. Elias, Mário Boieiro, Maria Teresa Ferreira, Paulo A. V. Borges

659C3462-F448-5934-A547-5A73A999D9B8 10.3897/BDJ.10.e80088.suppl10 Supplementary material 10 Trophic groups in Islands
Data type Figure Brief description Comparison of standardised abundance values species according to their different trophic groups: herbivores (A), predator (B), aprophyte (S). Different letters indicate significant differences between Islands, based on Dunn’s multiple comparison test (p < 0.05). FAI – Faial; FLO – Flores; PIC – Pico; SMG – São Miguel; SMR – Santa Maria; TER – Terceira. Fungivores are not represented as the group was represented by only two species and seven individuals present in three Islands (SMG, SMR, TER). File: oo_629376.docx https://binary.pensoft.net/file/629376 Noelline Tsafack, Rosalina Gabriel, Rui. B. Elias, Mário Boieiro, Maria Teresa Ferreira, Paulo A. V. Borges

F7BF9618-0738-55CE-8156-82DA809A31F7 10.3897/BDJ.10.e80088.suppl11 Supplementary material 11 Abundance proportion within functional groups for the different colonising status groups
Data type Figure Brief description Proportion of abundance per different functional groups at Archipelago (AZO) and Island level (FAI – Faial; FLO – Flores; PIC – Pico; SMG – São Miguel; SMR – Santa Maria; TER – Terceira) for all species (A), endemic (B), native (C) and introduced (D) species. S - saprophyte; P - predator; H - herbivore; F – fungivore. File: oo_658651.docx https://binary.pensoft.net/file/658651 Noelline Tsafack, Rosalina Gabriel, Rui. B. Elias, Mário Boieiro, Maria Teresa Ferreira, Paulo A. V. Borges

CA17D8A9-3D62-5CFE-BF36-A7F371C90058 10.3897/BDJ.10.e80088.suppl12 Supplementary material 12 Species richness within functional groups for the different colonising status groups
Data type Figure Brief description Proportion of species per different functional groups at Archipelago (AZO) and Island level (FAI – Faial; FLO – Flores; PIC – Pico; SMG – São Miguel; SMR – Santa Maria; TER – Terceira) for all species (A), endemic (B), native (C) and introduced (D) species. S - saprophyte; P - predator; H - herbivore; F – fungivore. File: oo_629378.docx https://binary.pensoft.net/file/629378 Noelline Tsafack, Rosalina Gabriel, Rui. B. Elias, Mário Boieiro, Maria Teresa Ferreira, Paulo A. V. Borges

DB26A83C-1834-5C59-9BA7-23982272C372 10.3897/BDJ.10.e80088.suppl13 Supplementary material 13 List of arthropod species associated with *Laurusazorica* in the Azores Data type Table Brief description The classification system follows the general guidelines presented in Borges et al. (2010), with the higher taxa listed in a phylogenetic sequence, from the less derived to more derived groups. The families, genera and species are listed by alphabetical order. The colonisation status of each species is presented in the 5th column of the list as follows: E – endemic; N – native; I – introduced. The functional group is given in the 6th column as follows: P – predator; H – herbivore; S – saprophyte; F – fungivore; Ex – External digestion and sucking; Ch – Chewing and cutting; Pi - Piercing and sucking; Si - Siphoning; No – Not feeding. X indicates species occurrence in the different Islands FAI – Faial; FLO – Flores; PIC – Pico; SMG – São Miguel; SMR – Santa Maria; TER – Terceira. Ten species identified only at the morphospecies level are not presented in the table. File: oo_629379.docx https://binary.pensoft.net/file/629379 Noelline Tsafack, Rosalina Gabriel, Rui. B. Elias, Mário Boieiro, Maria Teresa Ferreira, Paulo A. V. Borges

775A0468-EF7D-59F7-A5A8-02CF838128DF 10.3897/BDJ.10.e80088.suppl14 Supplementary material 14 Proportion of abundance within feeding mode groups for the different colonising status groups Data type Figure Brief description Proportion abundance per different feeding modes at Archipelago (AZO) and Island level (FAI – Faial; FLO – Flores; PIC – Pico; SMG – São Miguel; SMR – Santa Maria; TER – Terceira) for all species (A), endemic (B), native (C) and introduced (D) species. S - saprophyte; P - predator; H - herbivore; F – fungivore and Ex - external digestion and sucking; Ch - chewing and cutting; Pi - piercing and sucking; Si - siphoning. File: oo_629380.docx https://binary.pensoft.net/file/629380 Noelline Tsafack, Rosalina Gabriel, Rui. B. Elias, Mário Boieiro, Maria Teresa Ferreira, Paulo A. V. Borges

88F6E8D1-9D35-5048-8F3F-EAAA3229E77F 10.3897/BDJ.10.e80088.suppl15 Supplementary material 15 Proportion of species within feeding mode groups for the different colonising status groups Data type Figure Brief description Proportion of species per different feeding modes at Archipelago (AZO) and Island level (FAI – Faial; FLO – Flores; PIC – Pico; SMG – São Miguel; SMR – Santa Maria; TER – Terceira) for all species (A), endemic (B), native (C) and introduced (D) species. S - saprophyte; P - predator; H - herbivore; F – fungivore and Ex - external digestion and sucking; Ch - chewing and cutting; Pi - piercing and sucking; Si - siphoning. File: oo_629381.docx https://binary.pensoft.net/file/629381 Noelline Tsafack, Rosalina Gabriel, Rui. B. Elias, Mário Boieiro, Maria Teresa Ferreira, Paulo A. V. Borges

B114E57F-14A6-5C91-A52B-F9FCA07E7FAF 10.3897/BDJ.10.e80088.suppl16 Supplementary material 16 Proportions of rare species Data type Table Brief description Proportions of rare species (the first seven frequencies- Propf1f7), singleton species (represented by one individual Propf1), doubletons species (represented by two individuals-Propf2) and tripletons species (represented by three individual-Propf3). Proportions are calculated for all endemic, native and introduced species at the Archipelago level and in the six Islands (FAI – Faial; FLO – Flores; PIC – Pico; SMG – São Miguel; SMR – Santa Maria; TER – Terceira). File: oo_629382.docx https://binary.pensoft.net/file/629382 Noelline Tsafack, Rosalina Gabriel, Rui. B. Elias, Mário Boieiro, Maria Teresa Ferreira, Paulo A. V. Borges

A43C4115-153A-5CEA-B351-E7F187689DBE 10.3897/BDJ.10.e80088.suppl17 Supplementary material 17 Species abundance distribution histograms in Islands Data type Figure Brief description Species abundance distribution histograms for arthropods collected in six Islands (FAI – Faial; FLO – Flores; PIC – Pico; SMG – São Miguel; SMR – Santa Maria; TER – Terceira) of Azores Archipelago with predicted values of the gambin model (black dots) all species (1^st^ column.total), endemic (2^nd^ column.End), native (3^rd^ column.Nat) and introduced (4^th^ column.Int) species. Graphs of the same column are scaled equally for the Y axis. File: oo_629383.docx https://binary.pensoft.net/file/629383 Noelline Tsafack, Rosalina Gabriel, Rui. B. Elias, Mário Boieiro, Maria Teresa Ferreira, Paulo A. V. Borges

## Figures and Tables

**Table 1.** Summary of arthropod taxa associated with *L.azorica*. Colonising status, classes and orders with number of families, species and specimens are indicated.

**Colonising status** **Class**, Order	Families	Species	Specimens
**Endemic species**			
**Arachnida**			
Araneae	7	11	1783
**Insecta**			
Coleoptera	2	2	44
Hemiptera	4	9	1639
Lepidoptera	3	6	915
Microcoryphia	1	1	29
Neuroptera	1	1	106
Psocoptera	2	4	58
Trichoptera	1	1	3
**Native species**			
**Arachnida**			
Araneae	6	7	902
Opiliones	1	1	12
**Insecta**			
Blattaria	1	1	379
Coleoptera	2	2	35
Hemiptera	7	10	3316
Lepidoptera	2	3	88
Microcoryphia	1	1	15
Psocoptera	3	5	362
Thysanoptera	1	1	1
**Introduced species**			
**Arachnida**			
Araneae	8	11	402
Pseudoscorpiones	1	1	2
**Diplopoda**			
Julida	1	1	38
**Insecta**			
Coleoptera	2	2	5
Hemiptera	3	3	3
Lepidoptera	1	4	87
Psocoptera	2	2	79
Thysanoptera	2	4	10

**Table 2.** Total of number morphospecies and abundances for endemic (END), native non-endemic (NAT) and non-native introduced (INT) arthropods species associated with *L.azorica* in the six Islands (FAI – Faial; FLO – Flores; PIC – Pico; SMG – São Miguel; SMR – Santa Maria; TER – Terceira). Differences were assessed using a Chi-square test. Chi-square test estimates and significance are indicated.

	**Number of specimens**							**Number of species**				
	Total	END	NAT	INT	χ2	p	Total	END	NAT	INT	χ2	p
FAI	577	112	456	9	569.8	<0.0001	25	9	11	5	2.24	0.33
FLO	313	181	111	21	123.3	<0.0001	29	14	11	4	5.45	0.07
PIC	954	432	454	68	295.6	<0.0001	50	22	17	11	3.64	0.16
SMG	1202	705	463	34	576.4	<0.0001	52	21	19	12	2.58	0.28
SMR	855	202	544	109	368.3	<0.0001	38	15	13	10	1	0.61
TER	6412	2945	3082	385	2159.4	<0.0001	71	24	25	22	0.20	0.91
**Total**	**10313**	**4577**	**5110**	**626**	**3490.8**	**<0.0001**	**94**	**35**	**31**	**28**	**0.79**	**0.67**

**Table 3.** Distribution of rare species. Sample coverage (SC) and the first seven frequency counts (f1 ... f7) for all endemic, native and introduced species in the six Islands (FAI – Faial; FLO – Flores; PIC – Pico; SMG – São Miguel; SMR – Santa Maria; TER – Terceira). The first seven frequencies indicate the numbers of species represented by only 1, 2, 3, ... 7 individuals (singletons, doubletons, tripletons etc.) and Propf1f7 indicates the proportion of the sum of the first seven frequencies (i.e. proportion of rare species) to the total number of species (see the total number of species in Table 2).

	SC	f1	f2	f3	f4	f5	f6	f7	Propf1f7
**All species in the archipelago**									
Total_total	1	12	6	10	5	4	1	4	45
Total_Endemic	1	1	1	3	0	1	0	0	17
Total_Native	1	3	2	3	2	1	0	3	45
Total_Introduced	0.99	8	3	4	3	2	1	1	79
**All species in Islands**									
Total_FAI	0.98	11	5	0	1	1	0	2	80
Total_FLO	0.98	7	7	2	2	0	0	0	62
Total_PIC	0.98	17	3	5	2	1	1	0	58
Total_SMG	0.99	11	9	4	3	2	1	1	60
Total_SMR	0.99	6	5	5	4	1	0	0	55
Total_TER	1	12	5	4	5	2	4	1	46
**Endemic species in Islands**									
Endemic_FAI	0.96	4	1	0	1	0	0	0	67
Endemic_FLO	0.97	5	2	0	1	0	0	0	57
Endemic_PIC	0.99	6	2	3	1	0	0	0	55
Endemic_SMG	1	1	6	0	2	0	1	0	48
Endemic_SMR	1	1	2	1	2	1	0	0	47
Endemic_TER	1	0	1	1	1	2	0	0	21
**Native species in Islands**									
Native_FAI	0.99	3	4	0	0	0	0	1	73
Native_FLO	0.99	1	4	2	0	0	0	0	64
Native_PIC	0.99	6	0	0	1	1	1	0	53
Native_SMG	0.99	4	1	3	0	1	0	1	53
Native_SMR	1	1	3	2	1	0	0	0	54
Native_TER	1	3	1	1	2	0	4	1	48
**Introduced species in Islands**									
Introduced_FAI	0.59	4	0	0	0	1	0	0	100
Introduced_FLO	0.96	1	1	0	1	0	0	0	75
Introduced_PIC	0.93	5	1	2	0	0	0	0	73
Introduced_SMG	0.83	6	2	1	1	1	0	0	92
Introduced_SMR	0.96	4	0	2	1	0	0	0	70
Introduced_TER	0.98	9	3	2	2	0	0	0	73

**Table 4.** Alpha values and confidence intervals of species abundance distribution models for arthropods, collected on *L.azorica* in the Azores Archipelago. Values are given for all species, endemic, native and introduced species in the six Islands (FAI – Faial; FLO – Flores; PIC – Pico; SMG – São Miguel; SMR – Santa Maria; TER – Terceira).

	α-value	CI95_low	CI95_high
**All species in the Archipelago**			
Total_total	2.12	1.546	2.851
Total_Endemic	7.55	4.444	12.455
Total_Native	2.32	1.335	3.837
Total_Introduced	1.15	0.582	2.097
**All species in Islands**			
Total_FAI	0.69	0.303	1.386
Total_FLO	2.80	1.333	5.581
Total_PIC	1.48	0.857	2.436
Total_SMG	1.69	1.066	2.594
Total_SMR	2.21	1.296	3.608
Total_TER	2.19	1.497	3.136
**Endemic species in Islands**			
Endemic_FAI	1.40	0.264	5.437
Endemic_FLO	2.33	0.665	6.932
Endemic_PIC	2.19	0.934	4.734
Endemic_SMG	3.00	1.526	5.597
Endemic_SMR	9.90	3.791	24.923
Endemic_TER	11.84	5.973	22.764
**Native species in Islands**			
Native_FAI	1.041	0.330	2.658
Native_FLO	3.515	1.139	9.954
Native_PIC	1.991	0.735	4.696
Native_SMG	2.438	1.062	5.133
Native_SMR	2.657	1.043	6.102
Native_TER	2.779	1.471	4.947
**Introduced species in Islands**			
Introduced_FAI	0.41	0.006	3.264
Introduced_FLO	7.08	0.672	53.975
Introduced_PIC	1.29	0.285	4.590
Introduced_SMG	1.51	0.358	5.265
Introduced_SMR	1.40	0.3186	4.787
Introduced_TER	0.87	0.3583	1.846

**Table 5.** Dissimilarity analysis between Islands for arthropod species communities, associated with *L.azorica* using: all species(A), endemic (B), natives (C) and introduced (D) species. Values of Bray-Curtis dissimilarity index (lower half diagonal), number of shared species between the Islands (upper half diagonal) and number of species present in the Island (main diagonal in bold) are given. FAI – Faial; FLO – Flores; PIC – Pico; SMG – São Miguel; SMR – Santa Maria; TER – Terceira.

**(A)**	**FAI**	**FLO**	**PIC**	**SMG**	**SMR**	**TER**
**FAI**	**25**	14	19	21	23	13
**FLO**	0.84	**29**	25	22	17	24
**PIC**	0.8	0.67	**50**	40	29	41
**SMG**	0.87	0.78	0.66	**52**	26	46
**SMR**	0.78	0.7	0.65	0.74	**38**	33
**TER**	0.78	0.62	0.5	0.61	0.54	**71**

**(B)**	**FAI**	**FLO**	**PIC**	**SMG**	**SMR**	**TER**
**FAI**	9	5	8	8	5	8
**FLO**	0.77	14	11	10	8	10
**PIC**	0.78	0.71	22	18	13	18
**SMG**	0.91	0.85	0.75	21	12	17
**SMR**	0.76	0.73	0.64	0.79	15	13
**TER**	0.77	0.69	0.57	0.7	0.56	24

**(C)**	**FAI**	**FLO**	**PIC**	**SMG**	**SMR**	**TER**
**FAI**	11	7	8	9	6	11
**FLO**	0.86	11	9	8	7	10
**PIC**	0.77	0.59	17	14	10	15
**SMG**	0.81	0.64	0.52	19	10	17
**SMR**	0.77	0.62	0.63	0.66	13	11
**TER**	0.76	0.5	0.43	0.48	0.47	25

**(D)**	**FAI**	**FLO**	**PIC**	**SMG**	**SMR**	**TER**
**FAI**	5	2	3	4	2	5
**FLO**	0.96	4	4	4	2	4
**PIC**	0.75	0.84	11	8	6	8
**SMG**	0.88	0.91	0.72	12	4	8
**SMR**	0.83	0.88	0.76	0.86	10	9
**TER**	0.79	0.88	0.6	0.66	0.79	22
