# Supplementary material for: Arthropods and other biota associated with the Azorean trees and shrubs: Laurusazorica (Seub) Franco (Magnoliophyta, Magnoliopsida, Laurales, Lauraceae)
Source: Biodivers Data J. 2022 May 10;10:e80088. doi: 10.3897/BDJ.10.e80088 (PMC9848503; doi:10.3897/BDJ.10.e80088)
Supplement: Supplementary material 1 — List of references [file bdj-10-e80088-s001.docx]

List of references used in the survey of mosses, liverworts (bryophytes), and lichens associated with *Laurus azorica*

**BRYOPHYTES (mosses and liverworts)**

| **Nº** | **Citation** |
| --- | --- |
| 1 | Bates, J. W. & Gabriel, R. (1997). *Sphagnum cuspidatum* and *S. imbricatum* ssp. *affine* new to Macaronesia, and other new island records for Terceira, Azores. *Journal of Bryology*, 19 (3): 645-648. |
| 2 | Crundwell, A. C., Greven, H. C. & Stern, R. C. (1994). Some additions to the bryophyte flora of the Azores. *Journal of Bryology*, 18: 329-337. |
| 3 | Frahm, J.-P. (2004). A Guide to Bryological Hotspots in Europe. *Archives for Bryology*, 3: 4-14. |
| 4 | Gabriel, R. (1994). Briófitos da Ilha Terceira (Açores). *Ecologia, distribuição e vulnerabilidade de espécies seleccionadas*. M.Sc. thesis. Departamento de Ciências Agrárias. Universidade dos Açores. Angra do Heroísmo. |
| 5 | Mastracci, M. (2004). *Thamnobryum rudolphianum* (Neckeraceae, Musci), a new species from the Azores. *Lindbergia*, 29: 143-147. |
| 6 | Richards, P. W. (1937). A collection of bryophytes from the Azores. *Annales Bryologici*, 9: 131-138. |
| 7 | Sjögren, E. (1978). Bryophyte vegetation in the Azores Islands. *Memórias da Sociedade Broteriana*, 26: 1-273. |
| 8 | Sjögren, E. (1997). *Report on investigations of the bryoflora and bryovegetation in 1997 on the Azorean island of Terceira*. LIFE project. Departamento de Ciências Agrárias. Angra do Heroísmo. |
| 9 | SWEDISH MUSEUM OF NATURAL HISTORY (2006). *Moss Register*. (http://andor.nrm.se/fmi/xsl/kbo/publFinditems.xsl?-token.nav=items&-view&-db=kbo_mossregister&-token.languagecode=en-GB) |
| 10 | THE NEW YORK BOTANICAL GARDEN (2007). *C. V. Starr Virtual Herbarium*. (http://sweetgum.nybg.org/vh/specimen.php?irn=894690) |

**LICHENS**

| **Nº** | **Citation** |
| --- | --- |
| 1 | Aptroot, A. & Rodrigues, A. F. (2005a). Additions to the Azorean lichen flora. *Arquipélago. Life and Marine Sciences*, 22A: 71-75. |
| 2 | Aptroot, A. & Rodrigues, A. F. (2005b). New lichen records for the Azores, with the report of some tropical species new to Europe. *Cryptogamie-Mycologie*, 26 (3): 273-280. |
| 3 | Aptroot, A. (1989). Contribution to the Azores lichen flora. *Lichenologist*, 21 (1): 59-65. |
| 4 | Berger, F. & Aptroot, A. (2002). Further contributions to the flora of lichens and lichenicolous fungi of the Azores. *Arquipélago. Life and Marine Sciences*, 19A: 1-12. |
| 5 | Gabriel, R. & Bates, J. W. (2005). Bryophyte community composition and habitat specificity in the natural forests of Terceira, Azores. *Plant Ecology*, 177: 125–144. |
| 6 | HERBESS, 2007. *University Duisburg-Essen online lichen database*. Lichen Herbarium at the University Duisburg-Essen (HERBESS). 32 p. http://www.uni-essen.de/botanik/Pherb.htm |
| 7 | James, P. W. & White, F. J. (1987). Studies on the genus Nephroma 1. The European and Macaronesian species. *Lichenologist*, 19 (3): 215-268. |
| 8 | Purvis, O. W. & James, P. W. (1993). Studies on the lichens of the Azores. Part 1 - Caldeira do Faial. *Arquipélago. Life and Marine Sciences*, 11A: 1-15. |
| 9 | Purvis, O. W., James, & Smith, C. W. (1995). Studies on the lichens of the Azores. Part 3. Macrolichens of relict cloud forests. *Boletim do Museu Municipal do Funchal*, 4: 599-619. |
| 10 | Roux, C. & Sérusiaux, E. (2004). Le genre *Strigula* (lichens) en Europe et en Macaronésie. *Bibliotheca Lichenologica,* 90: 1-96 pp. |
