# Supplementary material for: Arthropods and other biota associated with the Azorean trees and shrubs: Laurusazorica (Seub) Franco (Magnoliophyta, Magnoliopsida, Laurales, Lauraceae)
Source: Biodivers Data J. 2022 May 10;10:e80088. doi: 10.3897/BDJ.10.e80088 (PMC9848503; doi:10.3897/BDJ.10.e80088)
Supplement: Supplementary material 2 — Vascular plants - division Pteridophyta [file bdj-10-e80088-s002.docx]

| Class | Order | Family | Taxa | Status |
| --- | --- | --- | --- | --- |
| Polypodiopsida | Hymenophyllales | Hymenophyllaceae | *Hymenophyllum tunbrigense* (L.) Sm. | NAT |
|  |  | Hymenophyllaceae | *Vandenboschia speciosa* (Willd.) G.Kunkel | NAT |
|  | Polypodiales | Dryopteridaceae | *Elaphoglossum semicylindricum* (T.E.Bowdich) Benl | MAC |
|  |  | Polypodiaceae | *Polypodium macaronesicum* subsp. *azoricum* (Vasc.) Rumsey, Carine & Robba | END |
