# Supplementary figures and images for: Arthropods and other biota associated with the Azorean trees and shrubs: Laurusazorica (Seub) Franco (Magnoliophyta, Magnoliopsida, Laurales, Lauraceae)
Source: Biodivers Data J. 2022 May 10;10:e80088. doi: 10.3897/BDJ.10.e80088 (PMC9848503; doi:10.3897/BDJ.10.e80088)

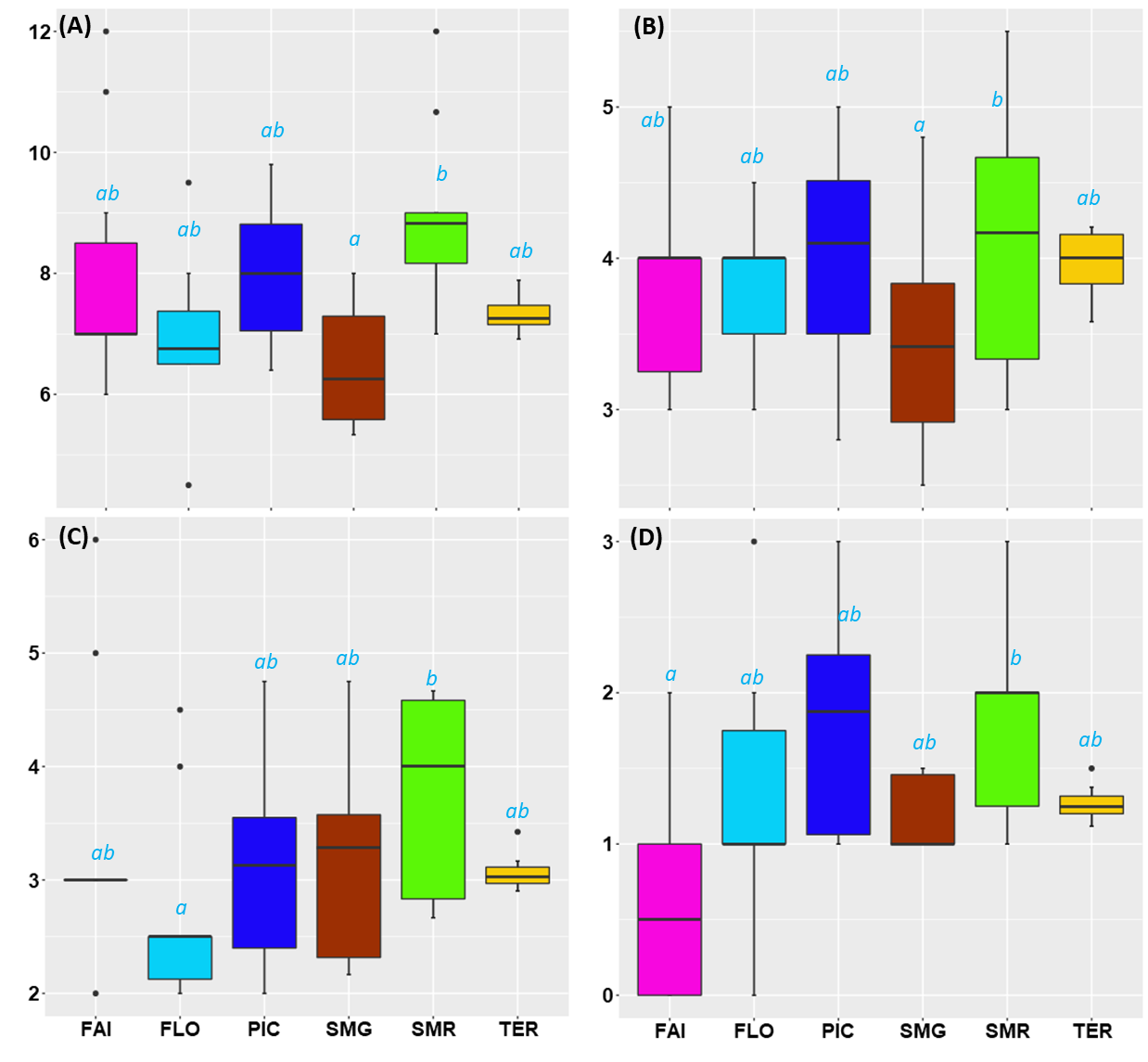

Supplement: Supplementary material 7 — Species richness in Islands [file bdj-10-e80088-s007.docx]

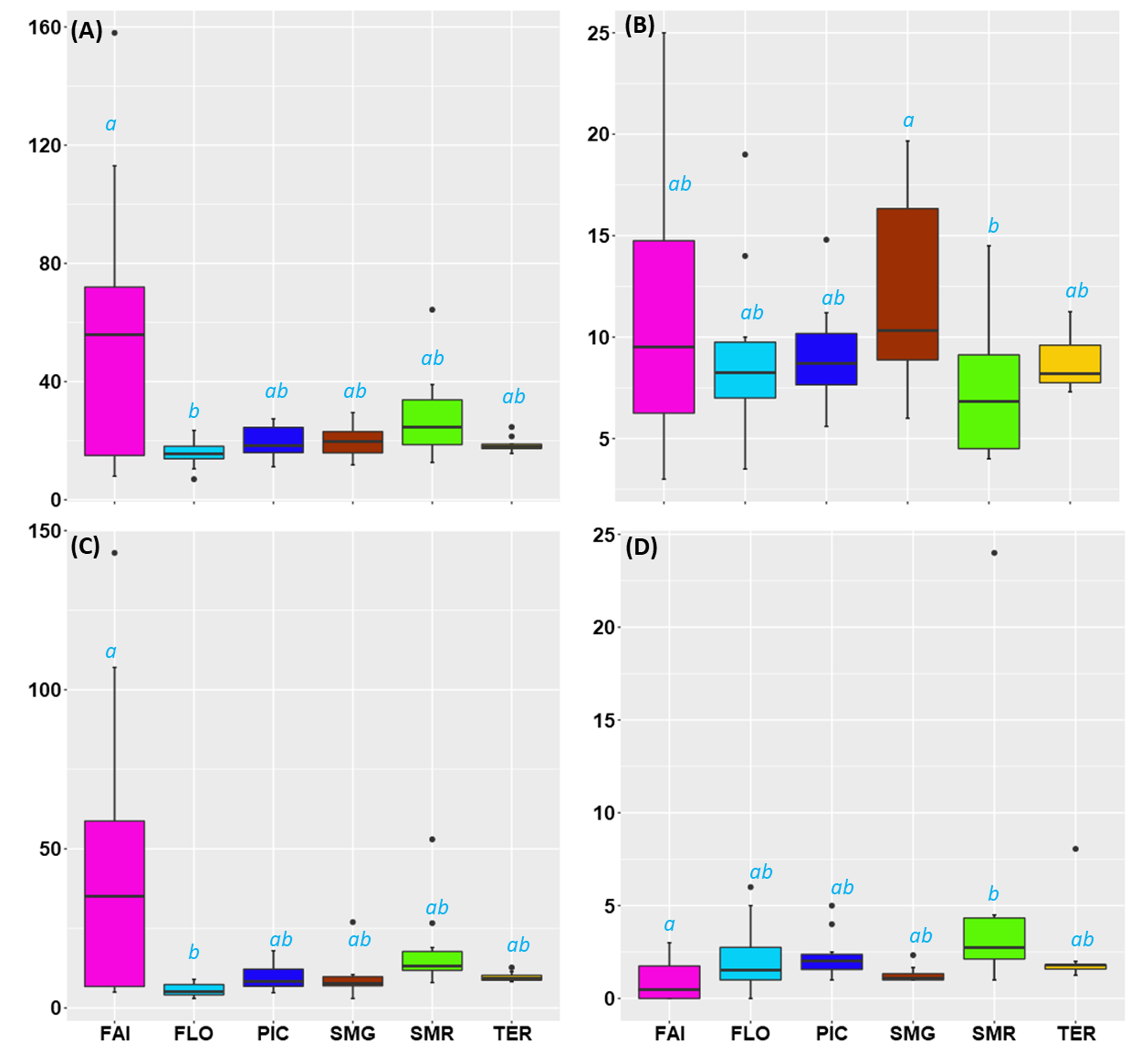

Supplement: Supplementary material 9 — Abundance in Islands [file bdj-10-e80088-s009.docx]

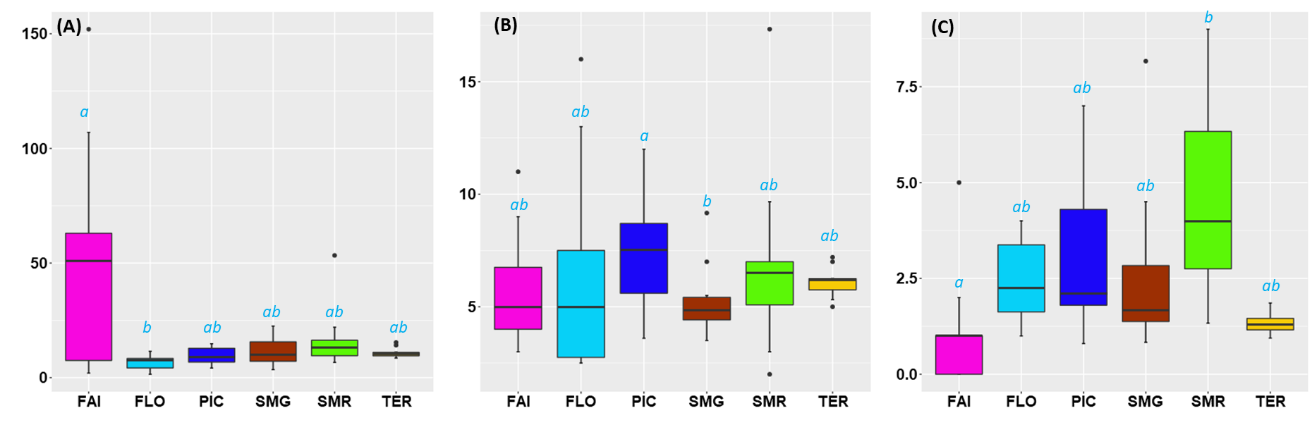

Supplement: Supplementary material 10 — Trophic groups in Islands [file bdj-10-e80088-s010.docx]

**
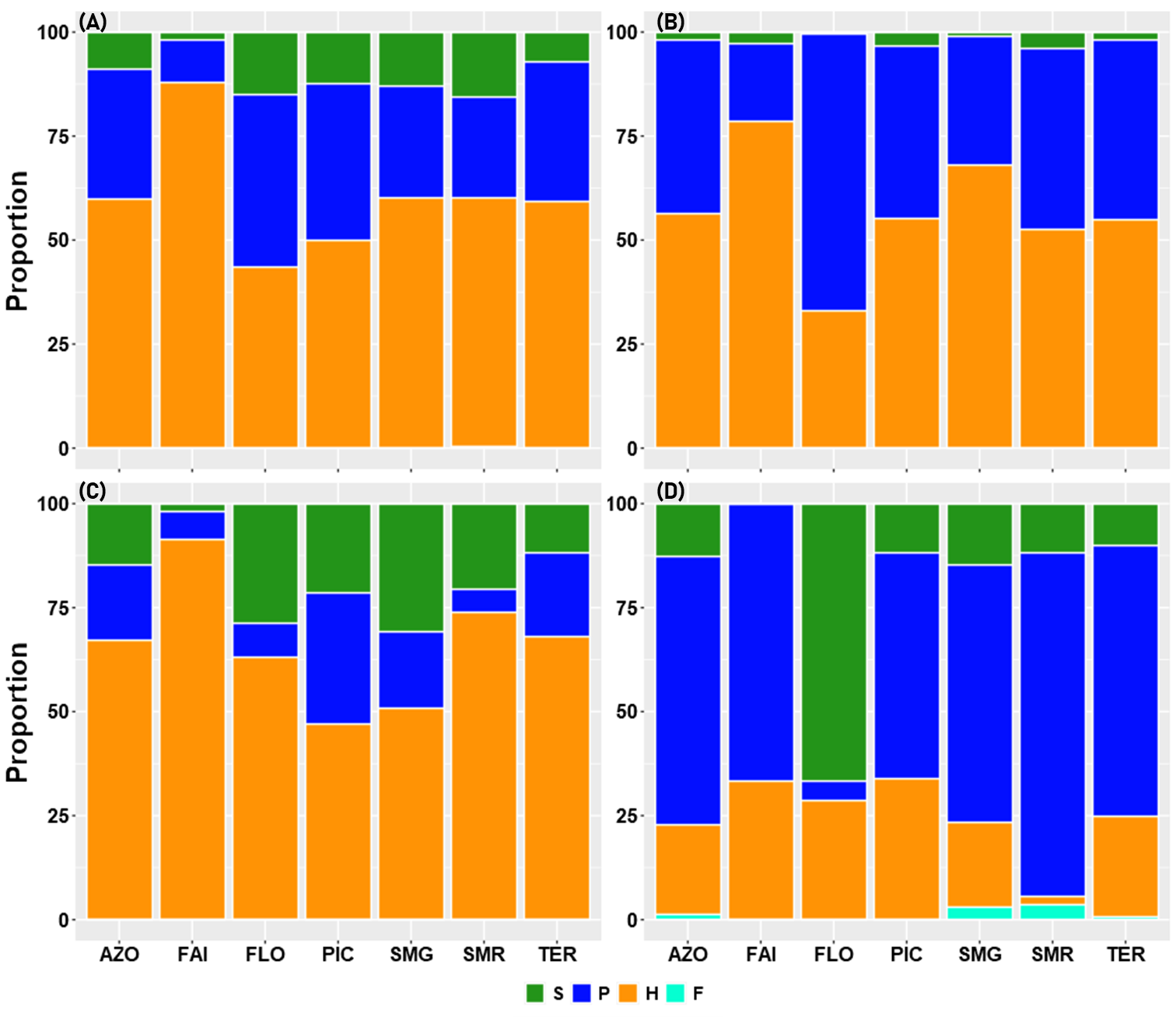
**

Supplement: Supplementary material 11 — Abundance proportion within functional groups for the different colonising status groups [file bdj-10-e80088-s011.docx]

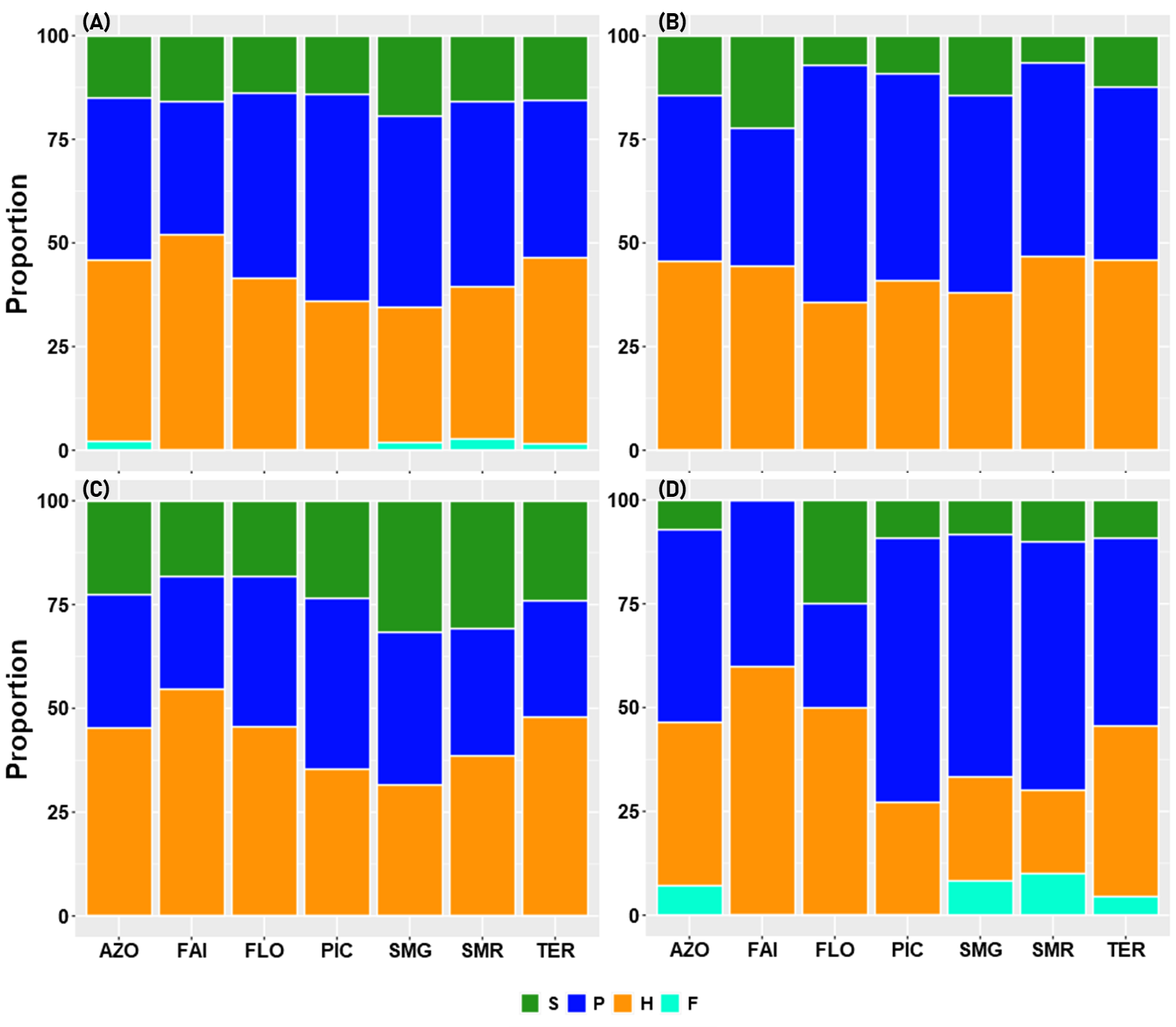

Supplement: Supplementary material 12 — Species richness within functional groups for the different colonising status groups [file bdj-10-e80088-s012.docx]

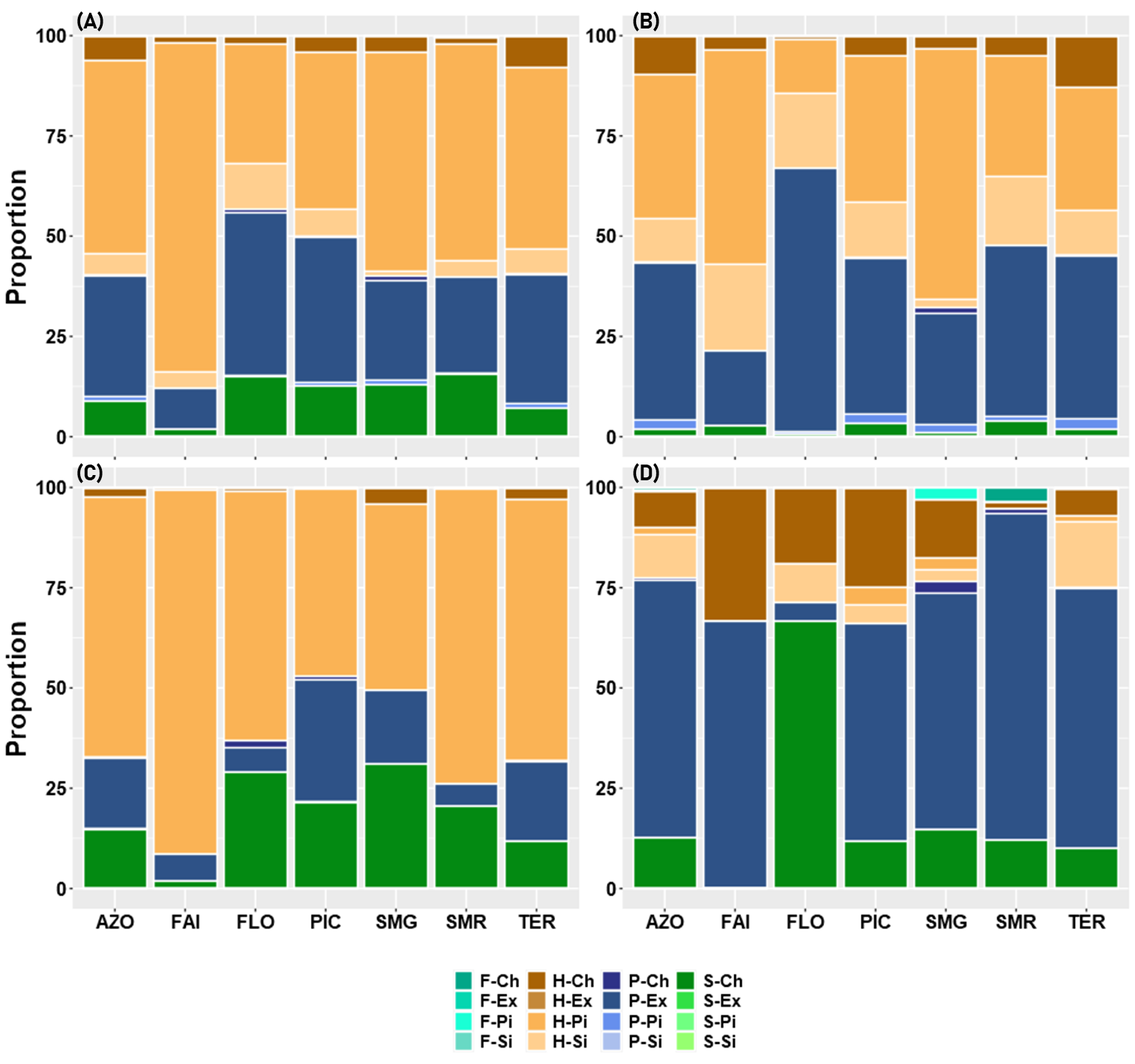

Supplement: Supplementary material 14 — Proportion of abundance within feeding mode groups for the different colonising status groups [file bdj-10-e80088-s014.docx]

**
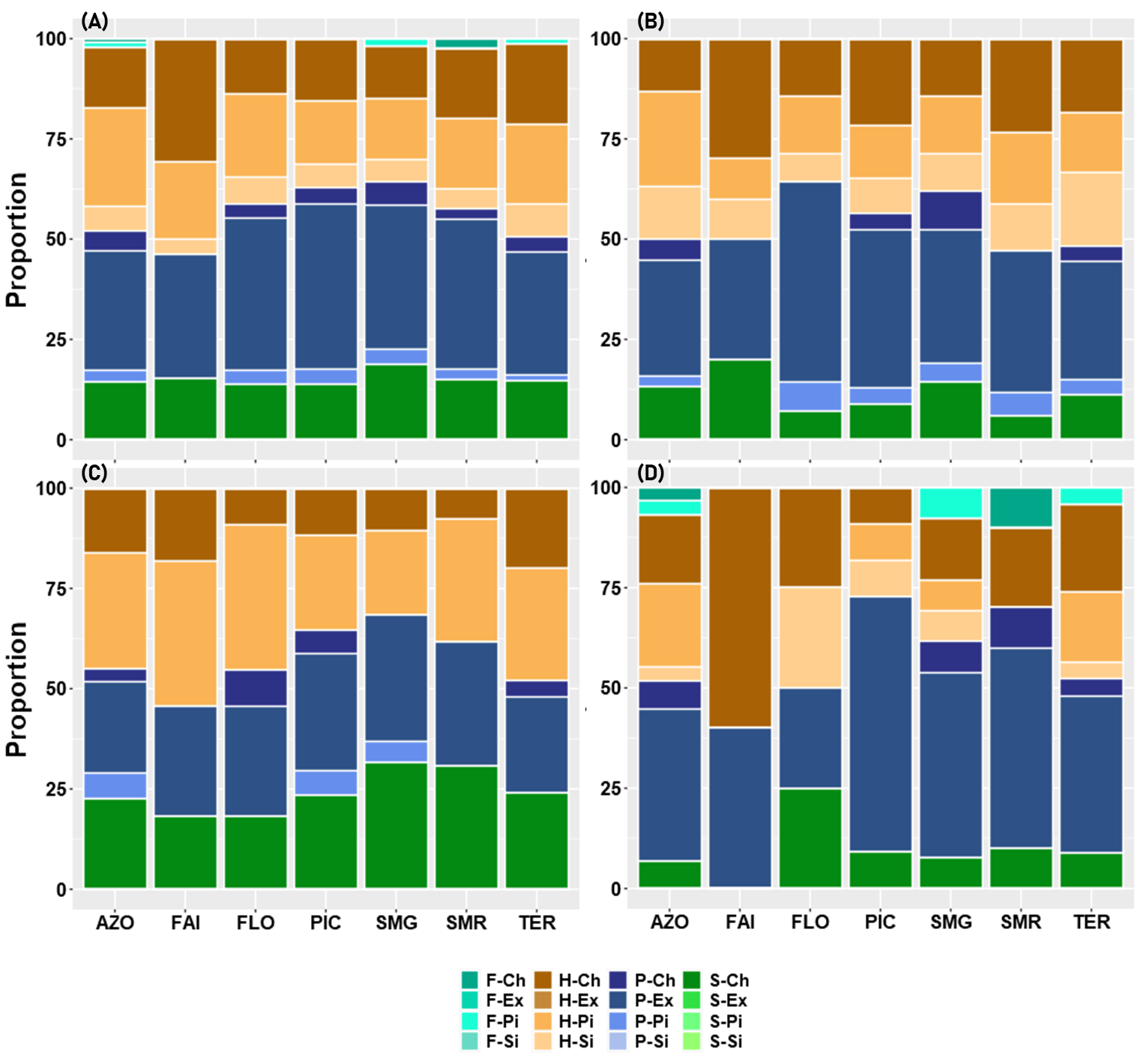
**

Supplement: Supplementary material 15 — Proportion of species within feeding mode groups for the different colonising status groups [file bdj-10-e80088-s015.docx]

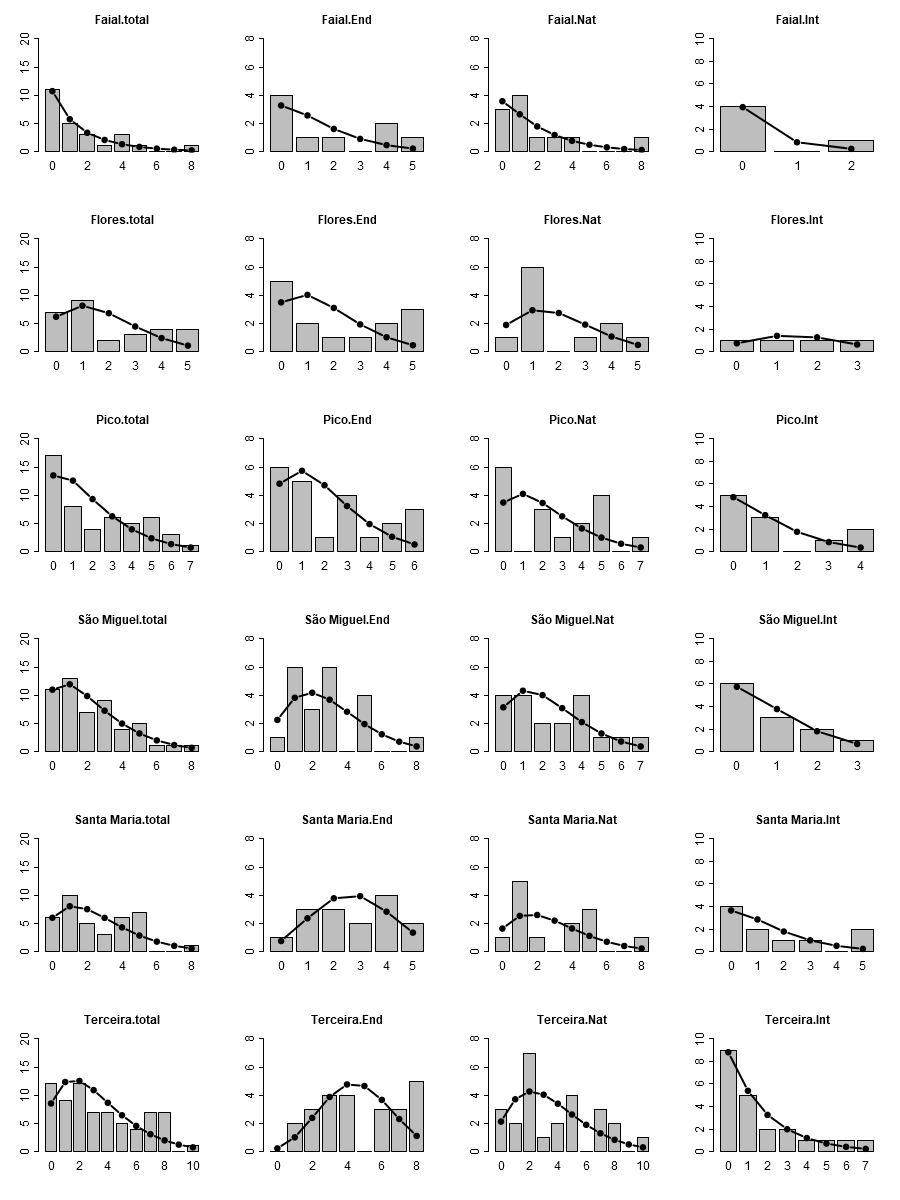

Supplement: Supplementary material 17 — Species abundance distribution histograms in Islands [file bdj-10-e80088-s017.docx]
