# Supplementary material for: Arthropods and other biota associated with the Azorean trees and shrubs: Laurusazorica (Seub) Franco (Magnoliophyta, Magnoliopsida, Laurales, Lauraceae)
Source: Biodivers Data J. 2022 May 10;10:e80088. doi: 10.3897/BDJ.10.e80088 (PMC9848503; doi:10.3897/BDJ.10.e80088)
Supplement: Supplementary material 8 — Standardised values of abundance in the different Islands [file bdj-10-e80088-s008.docx]

|  | **Number of specimens** | | | |  | **Number of species** | | | |
| --- | --- | --- | --- | --- | --- | --- | --- | --- | --- |
|  | Total | END | NAT | INT |  | Total | END | NAT | INT |
| Faial | 57.7 | 11.2 | 45.6 | 0.9 |  | 7.9 | 3.8 | 3.4 | 0.7 |
| Flores | 15.65 | 9.05 | 5.8 | 2.1 |  | 6.95 | 3.75 | 2.7 | 1.2 |
| Pico | 19.47 | 9.015 | 9.57 | 2.28 |  | 7.97 | 4 | 3.16 | 1.8 |
| SMG | 20.03 | 12.04 | 9.51 | 1.29 |  | 6.45 | 3.46 | 3.14 | 1.18 |
| SMR | 28.5 | 7.5 | 18.13 | 4.95 |  | 8.97 | 4.15 | 3.77 | 1.85 |
| TER | 18.61 | 8.70 | 9.69 | 2.33 |  | 7.32 | 3.96 | 3.07 | 1.26 |
