# Supplementary material for: Arthropods and other biota associated with the Azorean trees and shrubs: Laurusazorica (Seub) Franco (Magnoliophyta, Magnoliopsida, Laurales, Lauraceae)
Source: Biodivers Data J. 2022 May 10;10:e80088. doi: 10.3897/BDJ.10.e80088 (PMC9848503; doi:10.3897/BDJ.10.e80088)
Supplement: Supplementary material 13 — List of arthropod species associated with Laurusazorica in the Azores [file bdj-10-e80088-s013.docx]

| **Class** | **Order** | **Family** | **Species** | **Colonization Status** | **F. mode** | **FAI** | **FLO** | **PIC** | **SMG** | **SMR** | **TER** |
| --- | --- | --- | --- | --- | --- | --- | --- | --- | --- | --- | --- |
| Arachnida | Araneae | Araneidae | *Gibbaranea occidentalis* Wunderlich, 1989 | E | P-Ex | **x** | **x** | **x** | **x** | **x** | **x** |
|  |  |  | *Mangora acalypha* (Walckenaer, 1802) | I | P-Ex |  |  | **x** | **x** |  |  |
|  |  | Cheiracanthiidae | *Cheiracanthium erraticum* (Walckenaer, 1802) | I | P-Ex | **x** | **x** | **x** | **x** | **x** | **x** |
|  |  | Clubionidae | *Cheiracanthium floresense* Wunderlich, 2008 | E | P-Ex |  | **x** |  |  |  |  |
|  |  |  | *Clubiona terrestris* Westring, 1851 | I | P-Ex | **x** |  |  | **x** |  | **x** |
|  |  |  | *Porrhoclubiona decora* (Blackwall, 1859) | N | P-Ex |  |  | **x** | **x** | **x** | **x** |
|  |  | Dictynidae | *Nigma puella* (Simon, 1870) | I | P-Ex |  |  | **x** | **x** |  |  |
|  |  |  | *Lathys dentichelis* (Simon, 1883) | N | P-Ex | **x** | **x** | **x** | **x** | **x** | **x** |
|  |  | Linyphiidae | *Acorigone acoreensis* (Wunderlich, 1992) | E | P-Ex |  |  | **x** |  | **x** | **x** |
|  |  |  | *Minicia floresensis* Wunderlich, 1992 | E | P-Ex |  | **x** | **x** |  |  |  |
|  |  |  | *Savigniorrhipis acoreensis* Wunderlich, 1992 | E | P-Ex | **x** | **x** | **x** | **x** | **x** | **x** |
|  |  |  | *Walckenaeria grandis* (Wunderlich, 1992) | E | P-Ex |  |  |  |  |  | **x** |
|  |  |  | *Erigone atra* Blackwall, 1833 | I | P-Ex |  | **x** | **x** | **x** |  | **x** |
|  |  |  | *Mermessus bryantae* (Ivie & Barrows, 1935) | I | P-Ex |  |  |  |  |  | **x** |
|  |  |  | *Oedothorax fuscus* (Blackwall, 1834) | I | P-Ex |  |  |  |  |  | **x** |
|  |  |  | *Tenuiphantes tenuis* (Blackwall, 1852) | I | P-Ex |  |  | **x** | **x** | **x** | **x** |
|  |  |  | *Microlinyphia johnsoni* (Blackwall, 1859) | N | P-Ex |  |  |  | **x** |  | **x** |
|  |  |  | *Tenuiphantes miguelensis* (Wunderlich, 1992) | N | P-Ex |  |  |  |  | **x** | **x** |
|  |  | Lycosidae | *Pardosa acorensis* Simon, 1883 | E | P-Ex |  |  | **x** | **x** |  |  |
|  |  | Mimetidae | *Ero furcata* (Villers, 1789) | I | P-Ex |  |  | **x** |  | **x** | **x** |
|  |  | Pisauridae | *Pisaura acoreensis* Wunderlich, 1992 | E | P-Ex |  | **x** | **x** | **x** |  | **x** |
|  |  | Salticidae | *Macaroeris cata* (Blackwall, 1867) | N | P-Ex | **x** | **x** | **x** | **x** | **x** | **x** |
|  |  | Tetragnathidae | *Sancus acoreensis* (Wunderlich, 1992) | E | P-Ex | **x** | **x** | **x** | **x** | **x** | **x** |
|  |  |  | *Metellina merianae* (Scopoli, 1763) | I | P-Ex |  |  |  |  | **x** | **x** |
|  |  | Theridiidae | *Lasaeola oceanica* Simon, 1883 | E | P-Ex |  |  | **x** | **x** | **x** | **x** |
|  |  |  | *Rugathodes acoreensis* Wunderlich, 1992 | E | P-Ex |  | **x** | **x** | **x** | **x** | **x** |
|  |  |  | *Steatoda grossa* (C. L. Koch, 1838) | I | P-Ex |  |  | **x** |  | **x** | **x** |
|  |  |  | *Theridion musivivum* Schmidt, 1956 | N | P-Ex |  |  | **x** | **x** |  |  |
|  |  | Thomisidae | *Xysticus cor* Canestrini, 1873 | N | P-Ex | **x** | **x** | **x** | **x** |  | **x** |
|  | Opiliones | Phalangiidae | *Leiobunum blackwalli* Meade, 1861 | N | P-Ch | **x** | **x** |  |  | **x** |  |
|  | Pseudoscorpiones | Chthoniidae | *Ephippiochthonius tetrachelatus* (Preyssler, 1790) | I | P-Ch |  |  |  |  | **x** | **x** |
| Diplopoda | Julida | Julidae | *Ommatoiulus moreleti* (Lucas, 1860) | I | H-Ch | **x** | **x** | **x** | **x** | **x** | **x** |
| Insecta | Blattodea | Corydiidae | *Zetha simonyi* (Krauss, 1892) | N | S-Ch | **x** | **x** | **x** | **x** | **x** | **x** |
|  | Coleoptera | Corylophidae | *Sericoderus lateralis* (Gyllenhal, 1827) | I | P-Ch |  |  |  | **x** |  |  |
|  |  | Curculionidae | *Calacalles subcarinatus* (Israelson, 1984) | E | H-Ch |  | **x** | **x** | **x** | **x** | **x** |
|  |  |  | *Pseudophloeophagus tenax* Wollaston, 1854 | N | H-Ch | **x** |  |  |  |  | **x** |
|  |  | Scraptiidae | *Anaspis proteus* Wollaston, 1854 | N | H-Ch |  |  | **x** | **x** |  | **x** |
|  |  | Staphylinidae | *Notothecta dryochares* (Israelson, 1985) | E | P-Ch |  |  |  | **x** |  | **x** |
|  |  |  | *Atheta fungi* (Gravenhorst, 1806) | I | F-Ch |  |  |  |  | **x** |  |
|  | Hemiptera | Aphididae | *Rhopalosiphum rufiabdominale* (Sasaki, 1899) | I | H-Pl |  |  |  |  |  | **x** |
|  |  | Cercopidae | *Philaenus spumarius* (Linnaeus, 1758) | I | H-Pl |  |  |  | **x** |  |  |
|  |  | Cicadellidae | *Eupteryx azorica* Ribaut, 1941 | E | H-Pl |  |  |  |  |  | **x** |
|  |  | Cixiidae | *Cixius azofloresi* Remane & Asche, 1979 | E | H-Pl |  | **x** |  |  |  |  |
|  |  |  | *Cixius azomariae* Remane & Asche, 1979 | E | H-Pl |  |  |  |  | **x** |  |
|  |  |  | *Cixius azopifajo azofa* Remane & Asche, 1979 | E | H-Pl | **x** |  |  |  |  |  |
|  |  |  | *Cixius azopifajo azopifajo* Remane & Asche, 1979 | E | H-Pl |  |  | **x** |  |  |  |
|  |  |  | *Cixius azoterceirae* Remane & Asche, 1979 | E | H-Pl |  |  |  |  |  | **x** |
|  |  |  | *Cixius insularis* Lindberg, 1954 | E | H-Pl |  |  |  | **x** |  |  |
|  |  | Delphacidae | *Megamelodes quadrimaculatus* (Signoret, 1865) | N | H-Pl |  |  |  |  |  | **x** |
|  |  | Drepanosiphidae | *Anoecia corni* (Fabricius, 1775) | I | H-Pl |  |  |  |  |  | **x** |
|  |  | Flatidae | *Cyphopterum adcendens* (Herrich-Schäffer, 1835) | N | H-Pl | **x** | **x** | **x** | **x** | **x** | **x** |
|  |  | Lachnidae | *Cinara juniperi* (De Geer, 1773) | N | H-Pl | **x** |  |  | **x** |  | **x** |
|  |  | Lygaeidae | *Kleidocerys ericae* (Horváth, 1909) | N | H-Pl |  | **x** | **x** | **x** | **x** | **x** |
|  |  |  | *Scolopostethus decoratus* (Hahn, 1833) | N | H-Pl |  |  |  |  | **x** |  |
|  |  | Miridae | *Pinalitus oromii J.* Ribes, 1992 | E | H-Pl |  | **x** | **x** | **x** | **x** | **x** |
|  |  |  | *Campyloneura virgula* (Herrich-Schaeffer, 1835) | N | P-PI |  |  |  | **x** |  |  |
|  |  |  | *Monalocoris filicis* (Linnaeus, 1758) | N | H-Pl |  |  | **x** |  |  | **x** |
|  |  |  | *Polymerus cognatus* (Fieber, 1858) | N | H-Pl | **x** | **x** |  |  |  |  |
|  |  | Psyllidae | *Strophingia harteni* Hodkinson, 1981 | E | H-Pl |  |  | **x** | **x** | **x** | **x** |
|  |  | Triozidae | *Trioza laurisilvae* Hodkinson, 1990 | N | H-Pl | **x** | **x** | **x** | **x** | **x** | **x** |
|  | Lepidoptera | Crambidae | *Eudonia luteusalis* (Hampson, 1907) | E | H-Sl |  |  |  |  |  | **x** |
|  |  |  | *Scoparia coecimaculalis* Warren, 1905 | E | H-Sl |  |  | **x** | **x** |  | **x** |
|  |  | Geometridae | *Ascotis fortunata azorica* Pinker, 1971 | E | H-Ch | **x** | **x** | **x** | **x** |  | **x** |
|  |  |  | *Cyclophora azorensis* (Prout, 1920) | E | H-Ch |  |  | **x** |  | **x** | **x** |
|  |  |  | *Xanthorhoe inaequata* Warren, 1905 | E | H-Ch | **x** |  | **x** | **x** | **x** | **x** |
|  |  |  | *Orthonama obstipata* (Fabricius, 1794) | N | H-Ch | **x** |  | **x** | **x** | **x** | **x** |
|  |  |  | *Rhopobota naevana* (Hübner, [1817]) | I | H-Ch | **x** | **x** | **x** | **x** |  | **x** |
|  |  | Yponomeutidae | *Argyresthia atlanticella* Rebel, 1940 | E | H-Ch | **x** | **x** | **x** | **x** | **x** | **x** |
|  | Microcoryphia | Machilidae | *Trigoniophthalmus borgesi* Mendes, Gaju, Bach & Molero, 2000 | E | S-Ch |  |  |  |  |  | **x** |
|  |  |  | *Dilta saxicola* (Womersley, 1930) | N | S-Ch |  |  | **x** | **x** | **x** | **x** |
|  | Neuroptera | Hemerobiidae | *Hemerobius azoricus* Tjeder, 1948 | E | P-PI |  | **x** | **x** | **x** | **x** | **x** |
|  | Psocoptera | Caeciliusidae | *Valenzuela burmeisteri* (Brauer, 1876) | N | S-Ch |  |  |  |  | **x** |  |
|  |  |  | *Valenzuela flavidus* (Stephens, 1836) | N | S-Ch |  | **x** | **x** | **x** | **x** | **x** |
|  |  | Ectopsocidae | *Ectopsocus briggsi* McLachlan, 1899 | I | S-Ch |  | **x** | **x** | **x** | **x** | **x** |
|  |  | Elipsocidae | *Elipsocus azoricus* Meinander, 1975 | E | S-Ch | **x** |  | **x** | **x** |  | **x** |
|  |  |  | *Elipsocus brincki* Badonnel, 1963 | E | S-Ch | **x** |  | **x** | **x** |  | **x** |
|  |  | Lachesillidae | *Lachesilla greeni* (Pearman, 1933) | I | S-Ch |  |  |  |  |  | **x** |
|  |  | Trichopsocidae | *Trichopsocus clarus* (Banks, 1908) | N | S-Ch | **x** |  | **x** | **x** |  | **x** |
|  | Thysanoptera | Aeolothripidae | *Aeolothrips gloriosus* Bagnall, 1914 | N | P-PI |  |  | **x** |  |  |  |
|  |  | Phlaeothripidae | *Eurythrips tristis* Hood, 1941 | I | H-Pl |  |  |  |  |  | **x** |
|  |  |  | *Hoplothrips ulmi* (Fabricius, 1781) | I | F-PI |  |  |  | **x** |  | **x** |
|  |  | Thripidae | *Aptinothrips rufus* (Haliday, 1836) | I | H-Pl |  |  |  |  |  | **x** |
|  |  |  | *Heliothrips haemorrhoidalis* (Bouché, 1833) | I | H-Pl |  |  | **x** |  |  |  |
|  | Trichoptera | Limnephilidae | *Limnephilus atlanticus* Nybom, 1948 | E | P-Ch |  |  | **x** | **x** |  |  |
