# Supplementary material for: Arthropods and other biota associated with the Azorean trees and shrubs: Laurusazorica (Seub) Franco (Magnoliophyta, Magnoliopsida, Laurales, Lauraceae)
Source: Biodivers Data J. 2022 May 10;10:e80088. doi: 10.3897/BDJ.10.e80088 (PMC9848503; doi:10.3897/BDJ.10.e80088)
Supplement: Supplementary material 16 — Proportions of rare species [file bdj-10-e80088-s016.docx]

|  | Propf1 | Propf2 | Propf3 | Propf1f7 |
| --- | --- | --- | --- | --- |
| **All species in the archipelago** | | |  |  |
| Total_total | 13 | 6 | 11 | 45 |
| Total_Endemic | 3 | 3 | 9 | 17 |
| Total_Native | 10 | 6 | 10 | 45 |
| Total_Introduced | 29 | 11 | 14 | 79 |
| **All species in Islands** | | |  |  |
| Total_FAI | 44 | 20 | 0 | 80 |
| Total_FLO | 24 | 24 | 7 | 62 |
| Total_PIC | 34 | 6 | 10 | 58 |
| Total_SMG | 21 | 17 | 8 | 60 |
| Total_SMR | 16 | 13 | 13 | 55 |
| Total_TER | 17 | 7 | 6 | 46 |
| **Endemic species in Islands** | | |  |  |
| Endemic_FAI | 44 | 11 | 0 | 67 |
| Endemic_FLO | 36 | 14 | 0 | 57 |
| Endemic_PIC | 27 | 9 | 14 | 55 |
| Endemic_SMG | 5 | 29 | 0 | 48 |
| Endemic_SMR | 7 | 13 | 7 | 47 |
| Endemic_TER | 0 | 4 | 4 | 21 |
| **Native species in Islands** | | |  |  |
| Native_FAI | 27 | 36 | 0 | 73 |
| Native_FLO | 9 | 36 | 18 | 64 |
| Native_PIC | 35 | 0 | 0 | 53 |
| Native_SMG | 21 | 5 | 16 | 53 |
| Native_SMR | 8 | 23 | 15 | 54 |
| Native_TER | 12 | 4 | 4 | 48 |
| **Introduced species in Islands** | | |  |  |
| Introduced_FAI | 80 | 0 | 0 | 100 |
| Introduced_FLO | 25 | 25 | 0 | 75 |
| Introduced_PIC | 45 | 9 | 18 | 73 |
| Introduced_SMG | 50 | 17 | 8 | 92 |
| Introduced_SMR | 40 | 0 | 20 | 70 |
| Introduced_TER | 41 | 14 | 9 | 73 |
